# Microsporidian *Encephalitozoon hellem* inhibits host mitophagy by inducing ERAD to degrade BNIP3L

**DOI:** 10.1371/journal.ppat.1014078

**Published:** 2026-03-23

**Authors:** Ziyun Zou, Yibo Hu, Zhongxia Guan, Jiajing Chen, Qingyao Zhang, Yinze Han, Junyu Zhu, Chunxia Wang, Bing Han, Tian Li, Zeyang Zhou

**Affiliations:** 1 State Key Laboratory of Resource Insects, Southwest University, Chongqing, China; 2 Chongqing Key Laboratory of Microsporidia Infection and Control, Southwest University, Chongqing, China; 3 School of Basic Medical Sciences, Shandong University, Jinan, Shandong, China; 4 College of Life Sciences, Chongqing Normal University, Chongqing, China; University of California, San Diego, UNITED STATES OF AMERICA

## Abstract

Microsporidia are known intracellular pathogens that infect nearly all animals and deeply manipulate host mitochondrial homeostasis for survival. Here, we report a novel mechanism by which the human-pathogenic *Encephalitozoon hellem* modulates the mitophagy machinery of its host. We identified the secreted protein EhPTP4 as a key effector in disrupting selective degradation processes in the infected cells. EhPTP4 is found to localize within the nucleus of infected cells, where it induces increased expression of endoplasmic reticulum-associated degradation (ERAD) pathway components, including HSPA5, HERPUD1, and PDIA4. This induction enhances protein ubiquitination in host cells and leads to the degradation of BNIP3L, a critical regulator of mitophagy. Investigation into the molecular interaction network revealed that EhPTP4 interacts with host corepressor RCOR1 and histone H3. This interaction modulates histone acetylation, specifically at H3K14ac sites, thereby further influencing the expression of a key ERAD gene, HERPUD1. This study uncovers a sophisticated strategy by which microsporidia manipulates both ER stress response and the histone acetylation to suppress mitophagy. These findings provide new insights into the mechanisms of microsporidian pathogenesis.

## Author summary

Microsporidian parasites, known for their intricate manipulation of host cellular processes, have been found to employ novel mechanisms in disrupting mitochondrial homeostasis. This study investigates *Encephalitozoon hellem*, a human-pathogenic microsporidian, and its strategy to inhibit mitophagy, the cellular process responsible for degrading damaged mitochondria. The research reveals that *E. hellem* secretes a key effector protein, EhPTP4, which localizes within the nucleus of host cells. This protein enhances the expression of components related to the Endoplasmic Reticulum-Associated Degradation (ERAD) pathway, leading to increased degradation of BNIP3L, a crucial regulator of mitophagy. Without BNIP3L, the host’s ability to perform mitophagy is significantly impaired, potentially aiding the parasite’s survival. Furthermore, EhPTP4 interacts with RCOR1 and histone H3, influencing histone acetylation at specific sites (H3K14ac), which in turn affects the expression of ERAD-related genes. This dual regulation, via both ER stress pathways and epigenetic modifications, highlights a sophisticated strategy by *E. hellem* to suppress mitophagy. This study enhances our understanding of microsporidian pathogenesis and identifies potential therapeutic targets, offering insights into host-parasite interactions that could inform future treatments.

## Introduction

Microsporidia are a large group of obligate intracellular eukaryotic parasites infecting nearly all invertebrates and vertebrates, including humans and economically important animals [1,2]. Microsporidian impact extends beyond human health to agriculture and animal husbandry, resulting in substantial economic losses [2,3]. Currently, at least 200 genera and 1700 species have been identified [4,5]. Phylogenomic analyses have revealed that microsporidia are related to fungi [6], yet their genomes exhibit significant reductions, losing genes involved in tricarboxylic acid cycle, fatty acid beta-oxidation, lipid biosynthesis and other pathways [7]. These reductions highlight their evolutionary adaptation to an obligate parasitic lifestyle, making them tightly reliant on host cellular machinery [7–9].

The simplification of microsporidia metabolic pathways indicates their high dependence on host nutrients and close interaction with host organelles. Most recent studies have revealed that microsporidian *Encephalitozoon cuniculi* subverts host autophagy to promote their own growth [10]. Particularly, investigations have unveiled the intricate interactions between microsporidia and host mitochondria. For example, microsporidian *Encephalitozoon hellem* directly binds to host mitochondria via interactions between its sporoplasm surface protein 1 (EhSSP1) and host voltage-dependent anion channels on the outer mitochondrial membrane [11]. Besides, microsporidian infection disrupts host mitochondrial dynamics by promoting fragmentation via hijacking the dynamin 1-like protein (DRP1), a key regulator of mitochondrial fission [12]. Moreover, microsporidian *E. cuniculi* and *Vittaforma corneae* inhibit host cell apoptosis by up-regulating the expression of anti-apoptotic genes and suppressing the expression of pro-apoptotic genes [13]. *E. cuniculi* was also found to inhibit host apoptosis by suppressing the cleavage activation of caspase 3 and down-regulating the phosphorylation modification of p53 [14].

In addition to mitochondria, the endoplasmic reticulum (ER) is another organelle that is often significantly modulated by pathogens [15,16]. ER is responsible for the synthesis and processing of proteins and fatty acids. The ER-associated degradation (ERAD) pathway is a critical quality control mechanism responsible for identifying and eliminating misfolded or aberrant proteins through ubiquitination-proteasomal degradation. Beyond maintaining protein homeostasis, ERAD plays pivotal roles in regulating immunity, metabolism, apoptosis, and mitophagy [17–22]. Notably, this pathway has dual significance in host-pathogen interactions. It is not only a defense mechanism against pathogenic factors but also exploited by certain parasites to modulate cellular environments for their survival [23–27]. Moreover, ER establishes connections with mitochondria through mitochondrial-associated membrane domains (MAMs). ERAD precisely controls ER-mitochondria contact and the dynamic balance of mitochondria by regulating the homeostatic renewal of key MAMs proteins [28].

It remains unclear whether microsporidia exert cascading regulatory effects within the ER-mitochondria regulatory network of their hosts to facilitate their own growth and proliferation. In this study, we provide evidences that *E. hellem* inhibits host mitophagy by enhancing ERAD, leading to the degradation of BCL2 interacting protein 3-like (BNIP3L) through the action of polar tube protein 4 (EhPTP4). Notably, EhPTP4 has been previously implicated in facilitating infection by interacting with the host transferrin receptor 1 [29]. Our findings uncover a novel mechanism through which microsporidia manipulate the host mitochondrial quality control pathways, specifically by blocking mitophagy to ensure their survival within host cells.

## Results

### EhPTP4 is a secreted protein that enters the host nucleus

**Fig 1:**
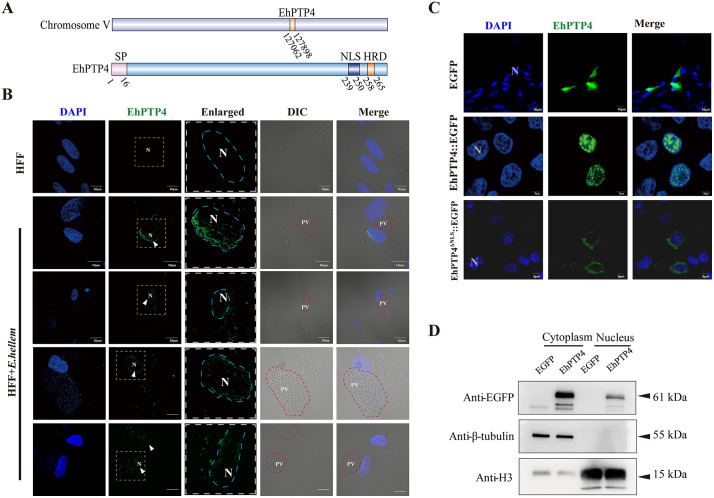
Sequence and secretory localization characteristics of EhPTP4. **(A)** Characteristics of the EhPTP4 sequence. SP, Signal peptide; NLS, Nuclear localization signal sequence; HRD, Histidine-rich domain. **(B)** HFF cells were infected with *E. hellem* for 48 hours. The cells were then fixed and immunostained using primary antibodies against EhPTP4, followed by an Alexa Fluor 488-conjugated anti-mouse-IgG secondary antibody (green). **(C)** HEK293 cells were transfected with the *EhPTP4::EGFP* and *EGFP* (control) for 48 hours, respectively. Autofluorescence of *EhPTP4::EGFP* and *EGFP* was observed (green). The nuclei (N) were stained with DAPI (blue). PV, Parasitophorous vacuole. **(D)** HEK293 cells were transfected separately with the *EhPTP4::EGFP* and *EGFP* (control) for 48 hours, respectively. Immunoblotting was performed to analyze the cytoplasmic and nuclear fractions. β-tubulin was used as a cytoplasmic marker, and histone H3 was used as a nuclear marker.

The protein EhPTP4 was initially identified as a component of the polar tube [29]. Domain analysis revealed that EhPTP4 contains both a signal peptide (SP) and a nuclear localization signal (NLS), suggesting its potential function as a secreted protein capable of targeting the host cell nucleus (**Fig 1A**). Moreover, the presence of a histidine-rich domain (HRD) in EhPTP4 implies possible involvement in transcriptional regulation within the nucleus [30,31]. To investigate the subcellular localization of EhPTP4 during infection, polyclonal antibodies were generated by immunizing mice with recombinant EhPTP4 protein. Immunofluorescence assays (IFA) showed that approximately 30% of infected cells exhibited an EhPTP4 signal in the nucleus (**Figs 1B**, **S1** **and** **S2**). Furthermore, plasmids encoding EhPTP4 and EhPTP4^ΔNLS^ fused to EGFP (EhPTP4::EGFP and EhPTP4^ΔNLS^::EGFP) were constructed and transfected into host cells, and IFA confirmed nuclear localization of the wild-type protein, but not the NLS-deleted variant (**Fig 1C**). Western blot analysis further validated these observations by detecting EhPTP4 in both nuclear and cytoplasmic fractions upon transfection (**Fig 1D**). Collectively, these findings strongly support that EhPTP4 is a secreted protein capable of localizing to the host cell nucleus, indicating its potential role in modulating host cellular processes during infection.

### EhPTP4 activates host ERAD pathway

**Fig 2:**
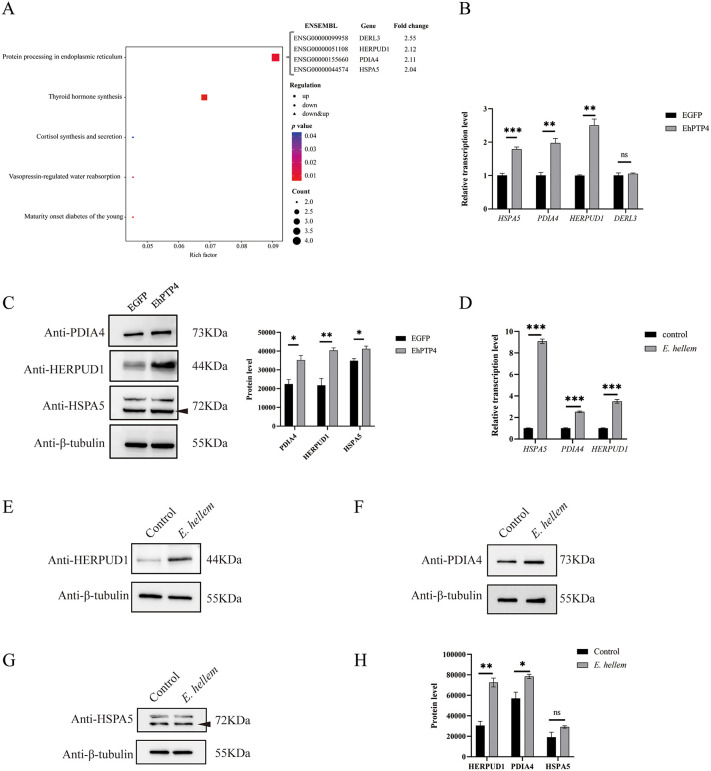
EhPTP4 upregulates gene expressions of host ERAD pathway. **(A)** HEK293 cells were separately transfected with plasmids *EhPTP4::EGFP* and *EGFP* (control) for 48 hours, respectively. KEGG enrichment analysis was performed on the differentially expressed genes (DEGs) obtained from the RNA-seq experiments. **(B)** HEK293 cells were transfected with *EhPTP4::EGFP* and *EGFP* (control) for 48 hours, respectively. Subsequently, the mRNA levels of ERAD-related genes (*HSPA5*, *PDIA4*, *HERPUD1*, and *DERL3*) were detected by RT-qPCR. **(C)** HEK293 cells were transfected with plasmids expressing EhPTP4::EGFP and EGFP (control) for 48 hours, respectively. Then, the protein levels of ERAD-related proteins (HSPA5, PDIA4, and HERPUD1) were detected by Western blot analysis. **(D)** HEK293 cells were infected with *E. hellem* for 48 hours. The mRNA levels of ERAD-related genes (*HSPA5*, *PDIA4* and *HERPUD1*) were detected by RT-qPCR. **(E)** HEK293 cells were infected with *E. hellem* for 48 hours. Then, the protein levels of HERPUD1 was detected by Western blot analysis. **(F)** HEK293 cells were infected with *E. hellem* for 48 hours. Then, the protein levels of PDIA4 were detected by Western blot analysis. **(G)** HEK293 cells were infected with *E. hellem* for 48 hours. Then, the protein levels of HSPA5 were detected by Western blot analysis. **(H)** The protein levels of E-G were detected by Western blot analysis. In panels B-H, the data are presented as the mean ± standard error of the mean (SEM) of three independent experiments. Statistical analysis was performed using Student’s t-test; ns indicates no significance; *, *p* < 0.05; **, *p* < 0.01; ***, *p* < 0.001.

To elucidate the role of EhPTP4 in *E. hellem* infection, HEK293 cells were transfected with *EhPTP4*, followed by RNA-seq analysis to assess transcriptional changes. Differentially expressed genes (DEGs) were identified using a threshold of fold change >2 and p-value <0.01, revealing 22 up-regulated and 12 down-regulated genes. Notably, KEGG pathway enrichment analysis indicated significant enrichment in the ERAD pathway, involving key components including HSPA5, PDIA4, HERPUD1, and DERL3 (**Fig 2A**). To further investigate the regulatory effects of EhPTP4 on ERAD-associated genes, RT-qPCR and Western blot analyses were performed, which revealed marked increases in both mRNA and protein levels of HSPA5, PDIA4, and HERPUD1 in EhPTP4-transfected cells (**Fig 2B and 2C**). Furthermore, RT-qPCR analysis of *E. hellem*-infected HEK293 cells confirmed the upregulation of HSPA5, HERPUD1, and PDIA4 (**Fig 2D**), and Western blot analysis during infection validated increased protein expression of HERPUD1 and PDIA4 (**Fig 2E–2H**). Collectively, these results strongly support that EhPTP4 modulates the expression of host genes involved in the ERAD pathway.

### EhPTP4 interacts with RCOR1 to regulate ERAD gene expression

**Fig 3:**
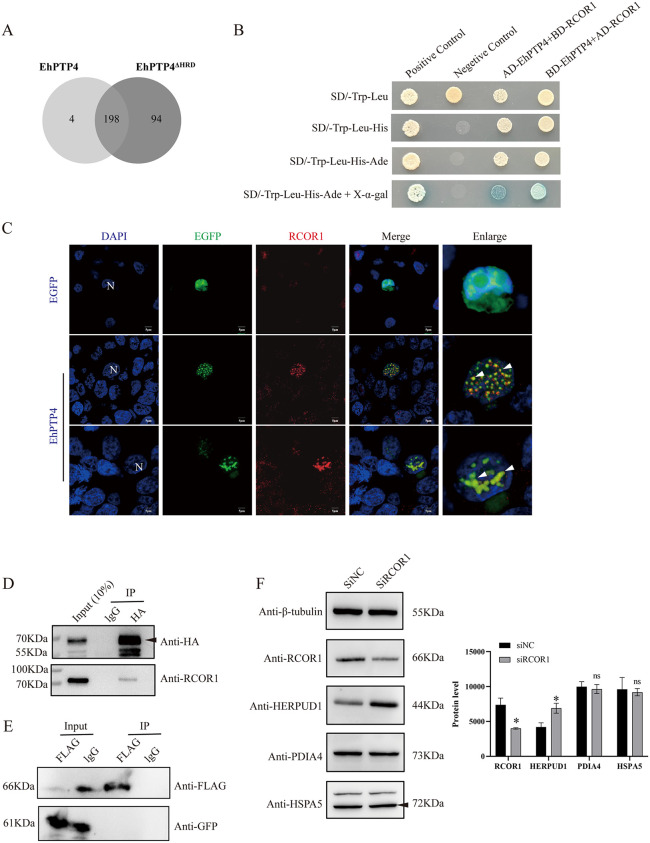
Regulation of candidate target proteins on the gene expression of the ERAD pathway. **(A)** Venn diagram showing the proteins enriched in EhPTP4 and EHPTP4^ΔHRD^. **(B)** The interaction between EhPTP4 and RCOR1 was detected using yeast two-hybridization. Y2H Gold cells were co-transfected with AD-EhPTP4 and BD-RCOR1. Subsequently, their growth was examined on SD/-Trp/-Leu, SD/-Trp/-Leu/-His, and SD/-Trp/-Leu/-His/-Ade plates. **(C)** HEK293 cells were transfected with *EhPTP4::EGFP* and *EGFP* (control) for 48 hours, respectively. The autofluorescence of EhPTP4::EGFP (green) was observed. The nuclei were stained with DAPI (blue). The nuclei without green fluorescent DAPI staining belong to untransfected cells, and only show the endogenous level of RCOR1 (weak red signal). The cells were fixed and immunostained with primary antibodies against RCOR1, followed by incubation with a secondary antibody, Alexa Fluor 647-conjugated anti-mouse-IgG (red). The white arrows indicate co-localization signals. **(D)** HEK293 cells were co-transfected with *EhPTP4::HA::EGFP* and *RCOR1::FLAG* for 48 hours. Then, immunoprecipitation (IP) was performed using protein G beads conjugated with anti-HA or anti-IgG (control), respectively. The Input and IP precipitates were analyzed via immunoblotting with the indicated antibodies against HA and RCOR1, respectively. **(E)** HEK293 cells were co-transfected with *EhPTP4*^*ΔHRD*^*::HA::EGFP and* *RCOR1::FLAG* for 48 hours. Then, immunoprecipitation (IP) was performed using protein G beads conjugated with anti-FLAG and anti-IgG (control), respectively. The Input and IP precipitates were analyzed via Western blot with anti-FLAG and anti-GFP, respectively. **(F)** HEK293 cells were transfected with siNC and siRCOR1 for 48 hours, respectively. Subsequently, the protein levels of RCOR1 and ERAD-related proteins (HERPUD1, PDIA4, and HSPA5) were detected by Western blot analysis.

To investigate the molecular mechanism by which EhPTP4 regulates host gene expression, we aimed to identify potential host cellular interacting partners of EhPTP4. Given that EhPTP4 contains a histidine-rich domain (HRD), which has been previously associated with transcriptional elongation [32], we hypothesize that EhPTP4 may modulate host gene expression through an HRD-dependent mechanism within the nucleus. To map the EhPTP4 interactome, we employed an APEX2-based biotin proximity labeling approach, a method that enables precise identification of proteins in close spatial proximity to a target protein via the biotin-streptavidin system [33]. HEK293 cells were transfected with either *EhPTP4* fused to *APEX2* (*EhPTP4::APEX2*) or an *HRD*-truncated variant (*EhPTP4*^*ΔHRD*^*::APEX2*). Immunofluorescence and Western blot analyses confirmed robust biotinylation of nuclear proteins in proximity to EhPTP4 (**S3 Fig**). Subsequent mass spectrometry analysis identified four specific candidate proteins (GM2A, CNTNAP1, NCCRP1, and RCOR1) that were significantly enriched exclusively in cells expressing EhPTP4::APEX2 (**Fig 3A** and **S1 Table**).

Subcellular localization analysis based on the UniProt database revealed that GM2A is localized in the lysosome, CNTNAP1 at the cell membrane, and NCCRP1 in the cytoplasm, whereas RCOR1 is specifically localized in the nucleus. This nuclear localization of RCOR1 is consistent with our research focus on transcriptional regulation, thereby prompting the selection of RCOR1 for further experimental validation. The interaction between EhPTP4 and RCOR1 was initially identified through a yeast two-hybrid assay (**Fig 3B**). Immunofluorescence analysis in HEK293 cells transfected with EhPTP4::EGFP demonstrated co-localization of EhPTP4::EGFP with RCOR1 within the nucleus (**Fig 3C**). Furthermore, co-immunoprecipitation assays confirmed the physical interaction between the two proteins (**Fig 3D**). To investigate the functional role of the HRD in this interaction, we generated an HRD deletion mutant (EhPTP4^ΔHRD^::EGFP). Transfection and subsequent immunoprecipitation experiments showed that the mutant failed to interact with RCOR1 (**Fig 3E**), indicating that the HRD is essential for the interaction between EhPTP4 and RCOR1.

To explore the functional significance of this interaction, we examined the role of RCOR1 in regulating the expression of ERAD-related genes. RNAi-mediated knockdown of RCOR1 in HEK293 cells resulted in a significant upregulation of HERPUD1 (**Fig 3F**). These findings underscore the functional relevance of the EhPTP4-RCOR1 interaction in modulating the expression of ERAD-related genes.

### Upregulation of ERAD genes expression by EhPTP4

**Fig 4:**
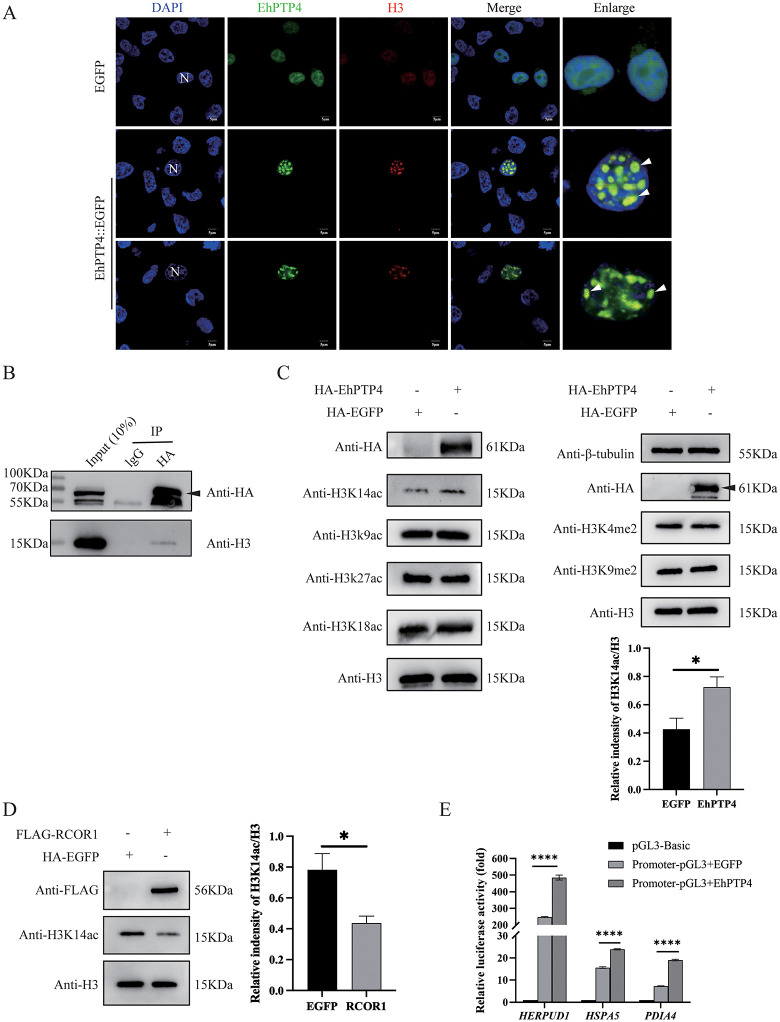
EhPTP4 interacts with host histone H3 and regulates H3K14ac. **(A)** HEK293 cells were transfected with *EhPTP4::EGFP* and *EGFP* (control) for 48 hours, respectively. Twelve *EhPTP4-*transfected cells were counted, among which nine (75%) showed positive signals for H3. The cell nuclei were stained with DAPI (blue). The cells were then fixed and immunostained using primary antibodies against H3, followed by a secondary antibody, Alexa Fluor 647-conjugated anti-mouse-IgG (red). The white arrows indicate the co-localization signals. **(B)** HEK293 cells were transfected with *EhPTP4::HA*. Subsequently, immunoprecipitation was performed using protein G beads. The input samples and immunoprecipitation precipitates were analyzed via Western blot with HA and H3 antibodies, respectively. **(C)** HEK293 cells were transfected with *EhPTP4::HA* for 48 hours. Subsequently, the protein levels of EhPTP4::HA and H3 were detected by Western blot with antibodies against HA and H3, respectively. Additionally, the acetylation and methylation of H3 were also detected by Western blot using specific antibodies, band intensities were quantified and normalized to H3. **(D)** HEK293 cells were transfected with *RCOR1::FLAG* for 48 hours. Subsequently, the protein levels of RCOR1::FLAG and H3 were detected by Western blot with antibodies against HA and H3, respectively. Additionally, the acetylation of H3K14 were also detected by Western blot using specific antibodies, band intensities were quantified and normalized to H3. **(E)** *EhPTP4* or *EGFP* was co-transfected with pGL3 plasmid encoding luciferase and promoter of *HERPUD1*, *HSPA5* or *PDIA4* into HEK293 cells, respectively. The basic pGL3::*luciferase* plasmid was used as a negative control. The luciferase activity was detected and normalized to the pRL-SV40 activity. Statistical analysis was performed using the Student’s t-test. ns, not significant; ****, *p* < 0.0001.

RCOR1 is a core component of an epigenetic modification complex that includes histone deacetylases (HDAC1/2) and lysine-specific demethylase LSD1/KDM1A, with histone H3 lysine residues serving as its primary substrate [34,35]. Given this association, we hypothesized that EhPTP4 may interact with histone H3. To test this hypothesis, HEK293 cells were transfected with the *EhPTP4*. Subsequent IFA revealed nuclear colocalization of EhPTP4 and histone H3 (**Fig 4A**), and co-immunoprecipitation (Co-IP) experiments confirmed their physical interaction (**Fig 4B**). These findings suggest that EhPTP4 may modulate the acetylation of histone H3 to regulate gene expression. To investigate this potential mechanism, we examined the acetylation and methylation status of histone H3 in HEK293 cells transfected with *EhPTP4*. Our results showed a significant increase in H3K14 acetylation (H3K14ac) (**Fig 4C**), indicating that EhPTP4 may interfere with histone deacetylation, thereby altering chromatin structure. To further assess the involvement of RCOR1 in regulating H3K14 acetylation, we analyzed H3K14ac levels in HEK293 cells transfected with RCOR1 and observed a significant reduction (**Fig 4D**). To identify the specific genomic regions affected by this modification, the promoters of the three ERAD-related genes (HERPUD1, HSPA5, and PDIA4) were cloned into a pGL3-Basic luciferase reporter vector. These constructs were co-transfected with EhPTP4 into HEK293 cells, and promoter activity was measured using a dual-luciferase reporter system. The results demonstrated that EhPTP4 significantly enhanced the transcriptional activity of all three promoters (**Fig 4E**), suggesting that it promotes the expression of ERAD-related genes by increasing H3K14 acetylation within their promoter regions.

### ERAD-mediated host regulatory networks modulated by EhPTP4

**Fig 5:**
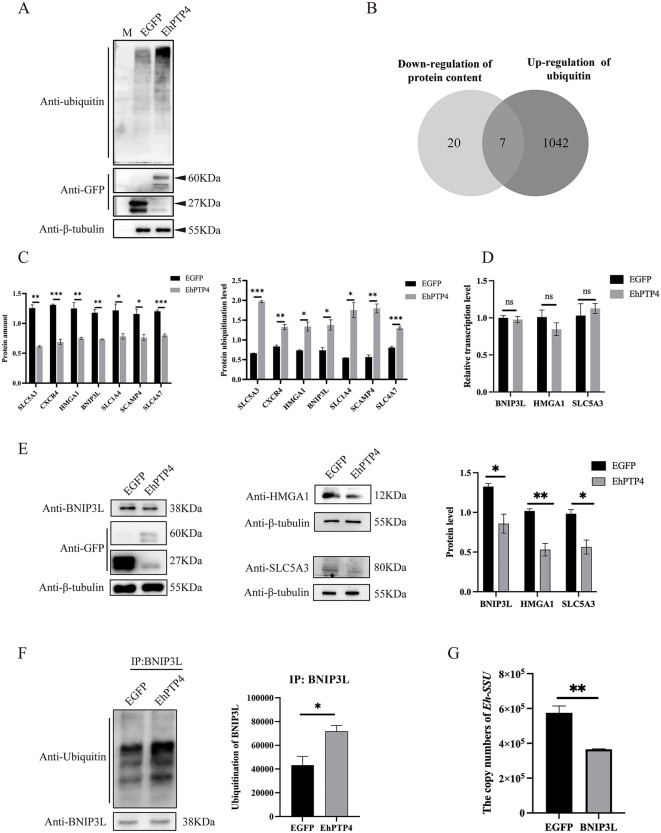
EhPTP4 promotes the host BNIP3L degradation via ubiquitin-proteasome system. **(A)** HEK293 cells were transfected with plasmids expressing EhPTP4 and EGFP (control) for 48 hours, respectively. Subsequently, Western blot analysis was performed to detect the ubiquitin levels, as well as the protein levels of GFP and tubulin. **(B)** Venn diagram depicting the upregulation of ubiquitination and downregulation of protein content. **(C)** Ubiquitin levels and protein levels of seven candidate proteins. **(D)** HEK293 cells were transfected with plasmids expressing EhPTP4 and EGFP (control) for 48 hours, respectively. RT-qPCR was then used to detect the mRNA levels of BNIP3L, HMGA1 and SLC5A3. **(E)** HEK293 cells were transfected with plasmids expressing EhPTP4 and EGFP (control) for 48 hours, respectively. Western blot analysis was then carried out to detect the ubiquitin levels, along with the protein levels of BNIP3L, HMGA1, SLC5A3, GFP, and tubulin. Band intensities were quantified and normalized to β-tubulin. **(F)** HEK293 cells were transfected with plasmids expressing EhPTP4 and EGFP (control) for 48 hours, respectively. The cells were lysed and used for immunoprecipitation experiments. The whole cell lysates (input) and immunoprecipitates were analyzed by Western blot with specific antibodies against BNIP3L and ubiquitin, respectively. **(G)** HEK293 cells were infected with *E. hellem* and transfected with plasmids expressing BNIP3L and EGFP (control) for 48 hours, respectively. Genomic DNA was extracted and copy numbers of *Eh-SSU* indicated the infection levels. In panels B-G, the data are presented as the mean ± standard error of the mean (SEM) of three independent experiments. Statistical analysis was performed using the Student’s t-test. ns indicates not significant; *, *p* < 0.05; **, *p* < 0.01; ***, *p* < 0.001.

EhPTP4 transfection induced a robust ERAD response, as evidenced by significantly elevated levels of protein ubiquitination (**Fig 5A**), indicating potential disruption of cellular protein homeostasis. To systematically investigate these alterations, quantitative proteomic and ubiquitinome analyses were performed in HEK293 cells transfected with *EhPTP4::EGFP* or *EGFP* control. This integrated approach identified a total of 27 proteins with reduced abundance, among which seven were hyper-ubiquitinated in the presence of EhPTP4 (**Fig 5B and** **S2** **and** **S3 Tables**). Additionally, 1,983 ubiquitination sites were mapped across 1,049 distinct proteins. Through comprehensive bioinformatics analysis integrating both proteome and ubiquitinome datasets, seven key proteins (BNIP3L, SLC5A3, CXCR4, HMGA1, SLC1A4, SCAMP4, and SLC4A7) were found to exhibit concurrent decreases in protein abundance and increases in ubiquitination levels (**Fig 5C**). Functional annotation revealed that these proteins are involved in critical cellular processes, including mitophagy, inositol metabolism, chemokine signaling pathways, DNA replication, and sulfur-containing amino acid metabolism. Our previous study demonstrated that *E. hellem* disrupts host mitochondrial function by inducing mitochondrial fragmentation, a process potentially linked to mitophagy [12]. Notably, BNIP3L, a well-characterized regulator of mitophagy [36], showed no change in transcript levels (**Fig 5D**), but displayed reduced protein abundance (**Fig 5E**) concomitant with increased ubiquitination (**Fig 5F**). Moreover, overexpression of BNIP3L significantly suppressed *E. hellem* proliferation (**Fig 5G**). Collectively, these results support the hypothesis that EhPTP4 suppresses host mitophagy by promoting ERAD-mediated ubiquitination and degradation of BNIP3L, thereby modulating mitochondrial homeostasis.

### EhPTP4 inhibits CCCP-induced host mitophagy

**Fig 6:**
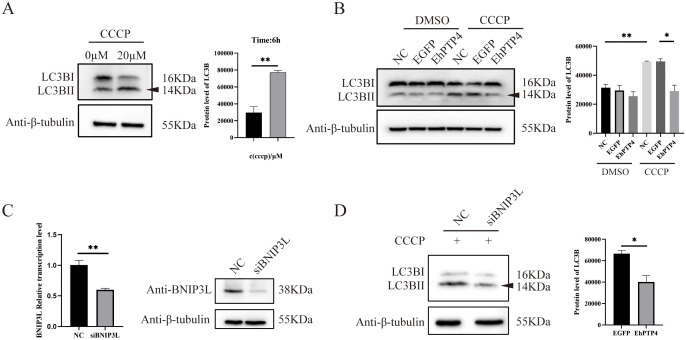
EhPTP4 inhibits host mitophagy induced by CCCP. **(A)** HEK293 cells were treated with CCCP (20 μM) for 6 hours. Western blot analysis was performed to detect the protein levels of tubulin (control), LC3I and LC3II, the latter of which indicates the activation of mitophagy. **(B)** HEK293 cells were transfected with plasmids expressing EhPTP4 and EGFP (control) for 48 hours, respectively. Subsequently, the cells were treated with CCCP (20 μM) for 6 hours. Western blot analysis was used to detect the protein levels of tubulin, LC3I and LC3II. **(C)** BNIP3L expression of HEK293 cells was knocked down by transfecting siBNIP3L for 48 hours. RT-qPCR was employed to detect the mRNA levels of BNIP3L, and Western blot was conducted to detect the protein levels of BNIP3L and tubulin (control). **(D)** HEK293 cells were transfected with siBNIP3L for 48 hours and then treated with CCCP (20 μM) for 6 hours. Western blot analysis was carried out to detect the protein levels of LC3I, LC3II and tubulin (control) using specific antibodies, respectively. The data are presented as the mean ± SEM of three independent experiments. Statistical analysis was performed using the Student’s t-test. *, *p* < 0.05; **, *p* < 0.01; ***, *p* < 0.001.

Carbonyl cyanide m-chlorophenyl hydrazone (CCCP), a proton-selective ion carrier, is widely used to induce mitochondrial depolarization and activate mitophagy by promoting the accumulation of mitophagy receptor proteins [37]. In the process of mitophagy, LC3BI and BII play a role in recognizing damaged mitochondria and targeting autophagy by binding to adaptor proteins such as p62 [38]. In this study, the HEK293 cells were treated with 20 μM CCCP for 6 hours to stimulate mitophagy. As expected, this treatment resulted in a significant increase of LC3BII activation, confirming successful induction of mitophagy (**Fig 6A**). To investigate the role of EhPTP4 in modulating this process, HEK293 cells expressing EhPTP4 were subjected to CCCP treatment. Western blot analysis revealed that LC3BII levels were substantially reduced in the presence of EhPTP4 compared to control conditions (**Fig 6B**), indicating a suppressive effect of EhPTP4 on mitophagy. To further explore the molecular mechanism underlying this inhibition, we examined the involvement of BNIP3L, a critical regulator of mitophagy. siRNA-mediated knockdown of BNIP3L significantly blocked CCCP-induced mitophagy (**Fig 6C and 6D**), suggesting that EhPTP4 probably promoted the degradation of BNIP3L by activating ERAD pathway so that inhibited the mitophagy.

### Protein levels of BNIP3L, p62, TOM20 in HEK293 cells infected by *E. hellem*

**Fig 7:**
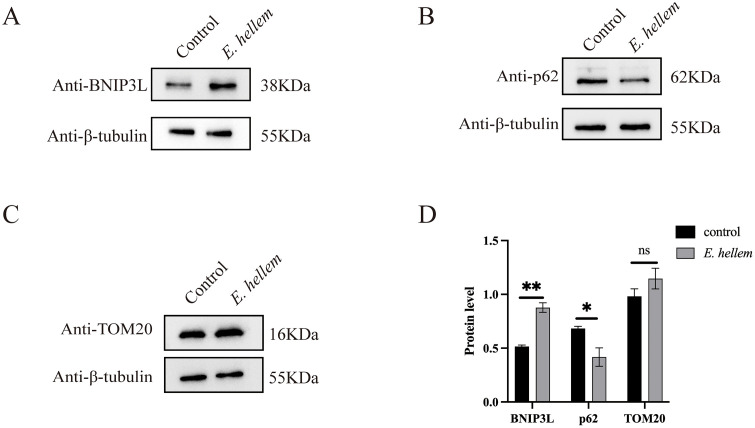
Western blot analysis on the protein levels of BNIP3L, p62 and TOM20. BNIP3L **(A)**, p62 (B) and TOM20 (C) in HEK293 cells after 48 hours of infection with *E. hellem*. **(D)** Densitometric analysis on the western blot of BNIP3L **(A)**, p62 (B) and TOM20 **(C)**. In panels D, band intensities were quantified and normalized to β-tubulin. The data are presented as the mean ± SEM of three independent experiments. Statistical analysis was carried out using the Student’s t-test. *, p < 0.05.**, p < 0.01.

In *E. hellem*-infected cells, BNIP3L significantly increased, while p62 significantly decreased (**Fig 7A and 7B**), indicating the induction of mitophagy upon infection. Furthermore, the mitochondrial homeostasis marker TOM20 was examined, and no significant change was observed (**Fig 7C and 7D**), suggesting that the overall mitochondrial mass remains in a state of homeostasis during infection.

### BNIP3L undergoes proteasomal degradation mediated by the ERAD pathway

**Fig 8:**
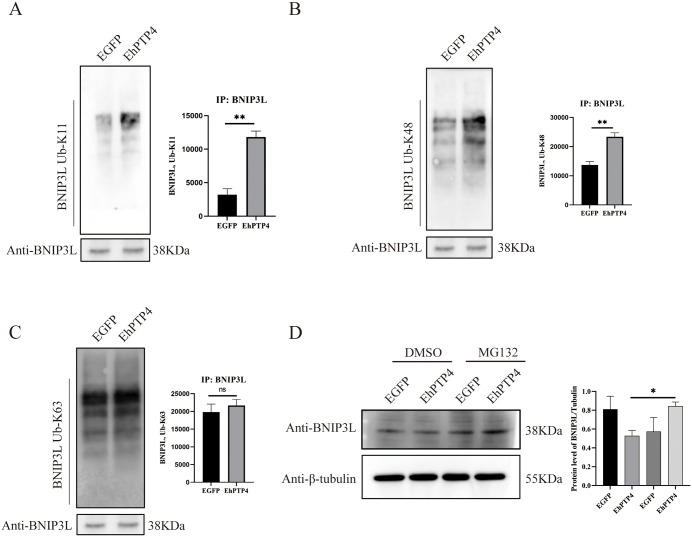
EhPTP4 promoted K11- and K48-Linked ubiquitination of BNIP3L. **(A-C)** HEK293 cells were transfected with plasmids expressing EhPTP4. Subsequently, the cell lysates were immunoprecipitated and analyzed via Western blot. **(D)** HEK293 cells were transfected with plasmids expressing EhPTP4 and EGFP (control) for 48 hours, respectively. Thereafter, the cells were treated with MG132 (20 nM) for 6 hours. The protein levels of BNIP3L and tubulin (control) were detected by Western blot, band intensities were quantified and normalized to β-tubulin. In panels A-D, the data are presented as the mean ± SEM of three independent experiments. Statistical analysis was carried out using the Student’s t-test. **, *p* < 0.01.

The above findings suggest that BNIP3L is likely subjected to degradation via the ERAD pathway activated by EhPTP4. To validate this hypothesis, we investigated the ubiquitination status of BNIP3L in HEK293 cells transfected with EhPTP4. Ubiquitination involves multiple types of lysine (K)-linked polyubiquitin chains (e.g., K6, K11, K27, K29, K33, K48, and K63), which are associated with distinct cellular fates such as proteasomal degradation (primarily mediated by K11 and K48 linkages) or signaling regulation (e.g., K6, K33, and K63 linkages) [39]. To determine the specific ubiquitination linkage types of BNIP3L, we performed Western blot analysis using linkage-specific antibodies. The results revealed a significant increase in K11- and K48-linked polyubiquitin chains on BNIP3L in EhPTP4-transfected cells, while no detectable changes were observed for K63-linked ubiquitination (**Fig 8A-8C**). These findings strongly indicate that BNIP3L undergoes ERAD-mediated proteasomal degradation in the presence of EhPTP4. To further confirm this conclusion, we treated the cells with MG132, a specific inhibitor of the 26S proteasome complex. Western blot analysis showed a marked accumulation of BNIP3L protein upon proteasome inhibition (**Fig 8D**), providing direct evidence that BNIP3L is degraded via the ERAD pathway in EhPTP4-transfected cells. Collectively, these experiments demonstrate that K11- and K48-linked polyubiquitination facilitates the degradation of BNIP3L through the ERAD-mediated proteasomal pathway. This discovery highlights a novel mechanism by which EhPTP4 regulates BNIP3L levels in cellular environments.

## Discussion

This research significantly advances our understanding of microsporidian pathogenesis by uncovering a previously unreported mechanism where a microsporidia-encoded protein manipulates both the ERAD pathway and mitochondrial quality control in host cells.

**Fig 9:**
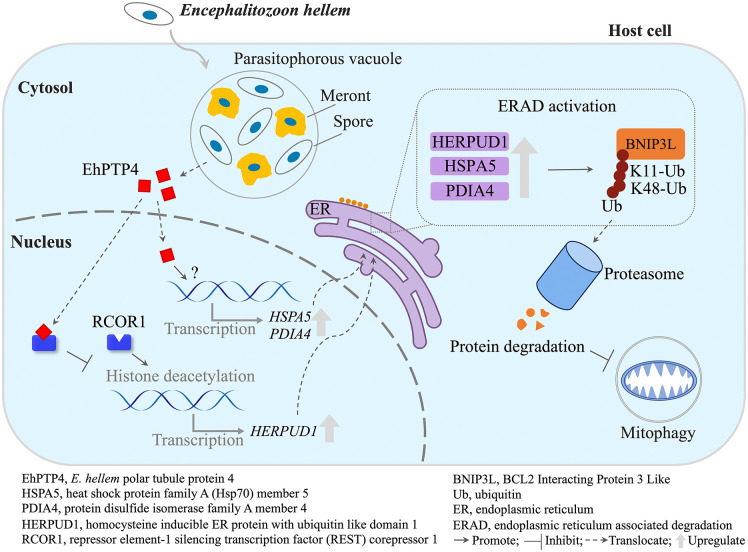
A model depicting how microsporidia promote the host ERAD to suppress mitophagy. *Encephalitozoon hellem* secretes the protein EhPTP4 into the host nucleus, which upregulates the expression of genes in the ERAD pathway (*HSPA5*, *HERPUD1*, and *PDIA4*). This, in turn, leads to the degradation of BNIP3L, a key protein involved in mitophagy, thereby inhibiting the host mitophagy defense mechanism.

Previously, EhPTP4 was known for its potential role in host cell infection as an important protein competent of the microsporidian polar tube [29]. This study expands our knowledge of its functional repertoire by demonstrating its ability to disturb host acetylation of H3 to hijack the ERAD pathway for interfering with the host defensive responses, including the mitophagy, a process essential for maintaining mitochondrial homeostasis, thereby facilitating microbial survival and persistence within the host (**Fig 9**). Moreover, microsporidia-induced ERAD-mediated protein degradation extends beyond BNIP3L to proteins involved in diverse cellular pathways such as inositol metabolism, chemokine signaling, DNA replication, and amino acid transport. This suggests that microsporidia likely hijack host ERAD to create a multi-faceted modulation of host cell metabolism, ensuring an optimal environment for its growth and reproduction. Microbial pathogens have evolved sophisticated strategies to manipulate host cellular processes, including epigenetic regulation, to establish a favorable environment for infection. The interaction of EhPTP4 with RCOR1 indicates that *E. hellem* probably disturbs host chromatin state, thus regulates gene expressions important for infection.

It is worth noting that multiple intracellular pathogens have also been reported to regulate host mitophagy and ER stress. For instance, Hepatitis B virus activates ERAD to reduce the accumulation of virus envelope proteins, which may promote the establishment of chronic infection by regulating the level of virus particles in infected cells [40]. Besides, Hepatitis C Virus (HCV) induces mitophagy through Parkin pathway to reduce the apoptosis of host cells [41]. *Listeria monocytogenes* induces oligomerization of mitophagy receptor NLRX1, promotes the binding of LIR motif of NLRX with LC3, and induces mitophagy [42]. These studies suggest that *E. hellem* probably prevents deacetylation of histone at *HERPUD1* gene region via a tethering of RCOR1 by EhPTP4, thus activates the expression of ERAD-related genes to potentially suppress host resistant response like mitophagy.

Notably, knockdown of RCOR1 resulted in an increase of *HERPUD1* but did not significantly alter the expression of other ERAD-associated genes like *PDIA4* and *HSPA5*. This suggests that EhPTP4 may employ multiple mechanisms to regulate ERAD pathway genes, possibly involving both chromatin remodeling and direct interaction with transcriptional regulators. These findings highlight the complexity of pathogen-driven chromatin remodeling and underscore the need for further investigation into the precise molecular interactions underlying this process.

Interestingly, *E. hellem* infection induces an overall increase in BNIP3L and a decrease in p62, suggesting activation of mitophagy. This is likely attributable to the fact that only a small proportion of infected cells exhibit nuclear localization of EhPTP4, resulting in mitophagy suppression being limited to a minor subset of cells. However, the expression level of TOM20 remains unchanged, indicating overall mitochondrial homeostasis in infected cells. Despite inducing mitophagy, the parasite concurrently triggers host mitochondrial fragmentation [12], which may contribute to maintaining mitochondrial equilibrium for balancing the reduction caused by mitophagy and apoptosis. Both mitophagy and apoptosis are known to restrict pathogen proliferation within host cells [43,44]. Previous studies have demonstrated that the microsporidian pathogens can inhibit host apoptosis [13,45]. Collectively, these results indicate that microsporidia modulate host mitochondrial morphology and function while preserving mitochondrial homeostasis to facilitate their intracellular proliferation.

In summary, this study challenges existing paradigms by demonstrating that microsporidia, despite their highly reduced genomes and lack of mitochondria, have evolved sophisticated molecular strategies to exploit host organelles and pathways. The identification of EhPTP4 as a multi-functional effector capable of hijacking ERAD and mitophagy pathways provide new insights into the evolutionary adaptations of these obligate intracellular parasites. Furthermore, this discovery opens novel avenues for exploring the interplay between microsporidian infection and host cellular quality control systems.

## Materials and methods

### Microsporidia and cell culture

*E. hellem* (ATCC 50451) was a gift from Prof. Louis Weiss (Albert Einstein College of Medicine, New York, USA) and was reproduced in rabbit kidney cells (RK13, ATCC CCL-37). HEK293 (ATCC, CRL-1573) and HFF (ATCC, CRL-2522) cells were cultured in Dulbecco’s modified Eagle Medium (DMEM) (Gibco/Thermo Fisher Scientific, C11995500BT) supplemented with 10% fetal bovine serum (FBS) (Gibco/Thermo Fisher Scientific, 10099158). All cells were incubated at 37°C in an environment with 5% CO_2_.

### Cell transfection

Plasmid transfection was carried out using Lipofectamine 3000 (Thermo Fisher Scientific Inc, L3000015) according to the manufacturer’s instructions. Forty-eight hours later, the transfected cells were collected to verify the expression and subcellular localization of the protein.

### Plasmid construction

The genes encoding *E. hellem* proteins were amplified from the *E. hellem* genomic DNA (gDNA) via the standard PCR procedure using PrimeSTAR Max Premix DNA polymerase (Takara, R045A). The DNA fragments conjugated to an HA tag and EGFP were then inserted into the pcDNA3.0 plasmid. Plasmids with deletion fragments were generated by overlapping PCR. The plasmid construct was verified through sequence analysis. HA-EGFP and EhPTP4-HA-EGFP sequence, which is inserted into pcDNA3.0 vector and uses CMV promoter, is a high copy plasmid, and the EGFP sequence does not contain NLS sequence.

### Antibodies

For Western blot analysis, the antibodies were commercially sourced and utilized in accordance with the manufacturer’s instructions. The antibodies included anti-β-tubulin (Beyotime, AT819; used at a dilution of 1:1000), anti-LC3B (Abcam, ab192890; 1:2000), anti-BNIP3L (Cell Signaling Technology, L12396; 1:1000), anti-CO-REST (Abcam, ab183711; 1:10000), anti-H3 (Abcam, ab1791; 1:5000), anti-HA (abmart, 26D11; 1:5000), anti-H3K9ac (Cell Signaling Technology, C5B11; 1:2000), anti-H3K14ac (Cell Signaling Technology, 7627; 1:2000), anti-H3K18ac (Beyotime, AG3829; 1:2000), anti-H3K27ac (Cell Signaling Technology, 8173; 1:2000), anti-Di-Methyl-Histone H3 (Lys4) (Cell Signaling Technology, 9725; 1:2000), anti-Di-Methyl-Histone H3 (Lys9) (Cell Signaling Technology, 4658; 1:2000), Anti-SQSTM1/p62 (Abcam, Ab109012; 1:5000), Anti-TOMM20 (Abcam, ab186735, 1:2000), Anti-HMGA1(Abcam, Ab129153; 1:1000), Anti-SLC5A3 (Abcam, ab110368, 1:500), anti-ubiquitin (PTM Bio, PTM1106; 1:1000), anti-Ub-k11 (ABclonal, A18197; 1:1000), anti-Ub-k48 (Abcam, ab140601; 1:1000), and anti-Ub-k63 (Abcam, ab179434; 1:1000). The following HRP-conjugated secondary antibodies were employed: HRP-labeled Goat Anti-Rabbit IgG (Sigma, 12–348; used at a dilution of 1:8000) and HRP-labeled Goat Anti-Mouse IgG (Sigma, 12–349; 1:8000). For immunofluorescence analysis, the antibodies of EhPTP4 were polyclonal antiserum prepared by intradermally inoculating mice with purified rEhPTP4 at a dosage of 100 μg per mouse.

### RNA-seq analysis

HEK293 cells were transfected with *HA::EGFP* and *EhPTP4::HA::EGFP* for 48 hours. The cells were first washed once with pre-cooled PBS, then digested with trypsin (Sangon Biotech, E607002) and subsequently collected. The collected cells were then flash-frozen in liquid nitrogen and stored at -80°C. RNA-seq analysis was performed on three replicates of each group by BioMarker (Beijing, China). Libraries were constructed and sequenced using the Illumina NovaSeq platform. Differentially expressed genes (DEGs) were analyzed using DEseq2, and only those detected in at least two replicates were deemed reliable for further analysis.

### Western blot analysis

Cell cultures were collected and washed three times with phosphate-buffered saline (PBS). Total cellular protein was extracted using RIPA neutral lysate (Beyotime, P0013B) supplemented with a proteasome inhibitor (MedChemExpress, HY-K0010) and a deubiquitinating enzyme (DUB) inhibitor (Beyotime, SG0020). The extracted proteins were boiled at 100°C after adding protein loading buffer, separated by sodium dodecyl sulfate-polyacrylamide gel electrophoresis (SDS-PAGE), and then transferred onto polyvinylidene difluoride (PVDF) membranes (sigma, 03010040001). These membranes were then incubated with a blocking solution (5% skim milk containing 0.05% Tween 20) for 2 hours at room temperature or overnight at 4°C. Subsequently, the PVDF membrane was incubated with primary antibodies for 2 hours and then washed three times with Tris-buffered saline with Tween 20 (TBST). Next, the PVDF membrane was incubated with goat anti-mouse IgG conjugated with horseradish peroxidase (HRP) or goat anti-rabbit IgG conjugated to HRP for 45 minutes at room temperature. After being washed three times with TBST, the membranes were developed using the ECL Western blot Detection Kit (Thermo Fisher Scientific, 34580) and imaged with the Azure Biosystems C300 imaging system.

### Indirect immunofluorescence assay (IFA)

Cells transfected in an orifice plate for 48 hours were washed three times with PBS, with each wash lasting 5 minutes. Subsequently, they were fixed with 500 μL of 4% paraformaldehyde (Biosharp, BL539A) for 12 minutes, followed by another three washes with PBS, each for 5 minutes. Then, 0.5% TritonX-100 (Sigma, 9036-19-5) was added to permeabilize the cells for 18 minutes, and the cells were washed three times with PBS again, each wash lasting 5 minutes. Next, the cells were blocked with 5% bovine serum albumin (M/V) + 10% sheep serum (V/V) at room temperature for 2 hours or at 4°C overnight. After diluting the primary antibody appropriately with PBS, it was incubated at room temperature for 2 hours or at 4°C overnight. The Alexa488 fluorescent secondary antibody was diluted 1:1000 in PBS and incubated at room temperature for 45 minutes. Streptavidin-AF (Alexa Fluor)-568, diluted 1:1000, was incubated simultaneously with the Alexa488 fluorescent secondary antibody in the dark. The diluted solution of 4’,6-diamidino-2-phenylindole (DAPI) (Sigma, D9542) was incubated at room temperature for 40 minutes. An anti-fluorescence quencher was used to prevent fluorescence quenching. Finally, the results were observed using a laser confocal microscope (Olympus FV1200).

### Quantitative analysis of proteome and ubiquitinated proteome

HEK293 cells were transfected with HA-EGFP (control) and EhPTP4-HA-EGFP for 48 hours. Subsequently, the cells were washed once with pre-cooled PBS, digested with trypsin, and then collected. The collected cells were immediately frozen in liquid nitrogen and stored at -80°C. RNA-seq analysis was performed on three replicates of each group. For ubiquitination analysis, the total ubiquitination level of the protein was first evaluated using Western blotting. Proteome and ubiquitome analyses were performed by PTM Bio (Hangzhou, China). The peptide was analyzed using a timsTOF Pro mass spectrometer.

### APEX2-mediated proximity labeling and mass spectrometry analysis

HEK293 cells were transfected with *EhPTP4::APEX2::HA* and *EhPTP4^ΔHRD^::APEX2::HA* expression constructs for 48 hours. After transfection, the cells were treated with DMEM containing biotin-phenol at 37°C for 30 minutes. Subsequently, oxidative crosslinking was induced by adding 1 mM H₂O₂ to the medium and incubating for 1 minute at room temperature. The treatment medium was discarded, and cells were immediately submerged in 5 mL of reaction termination solution (10 mM sodium azide, 10 mM sodium ascorbate, and 5 mM Trolox). Cells were then washed twice with 5 mL of reaction stop solution (5 min per wash), while negative control samples were washed twice with DPBS. Following the washing steps, cells were collected by centrifugation at 3000 × g for 5 minutes in 5 mL of reaction stop solution. The cell pellet was resuspended in RIPA neutral lysis buffer (106 cells/100 μL RIPA) supplemented with protease inhibitors (10 mM sodium azide, 10 mM sodium ascorbate, 5 mM Trolox, and 1 mM PMSF). The lysate was incubated on ice for 10 minutes to ensure complete cell lysis. After centrifugation at 12,000 × g for 5 minutes at 4°C, the supernatant containing solubilized proteins was collected. For affinity purification of biotinylated proteins, streptavidin magnetic beads were pre-equilibrated by washing twice with 1 mL RIPA neutral lysis buffer. Subsequently, 500 μL of protein lysate (5 mg total protein) was incubated with 80–100 µL of pre-washed streptavidin magnetic beads at room temperature for 1 hour to allow capture of biotinylabeled proteins. The bound complexes were washed twice with RIPA neutral lysis buffer supplemented with 1 M KCl and 0.1 M Na₂CO3 to elute non-specific binding proteins, followed by a final wash with 1 mL of RIPA buffer. Proteins bound to the magnetic beads were eluted by boiling in SDT buffer for 10 minutes, and the eluate was subjected to LC-MS/MS analysis. LC-MS/MS analysis was performed on a Q Exactive mass spectrometer (Thermo Scientific) interfaced with an Easy nLC chromatography system (Thermo Fisher Scientific).

### Immunoprecipitation

The transfected cell cultures were harvested and washed three times with PBS. Total proteins were extracted using RIPA neutral lysate (100 µL per 106 cells) containing a proteasome inhibitor and a deubiquitinase inhibitor. This was followed by centrifugation at 12000 g for 5 minutes. The supernatant was then incubated with 25 µL of Protein G and 25 µL of Protein A magnetic beads. Subsequently, the beads were washed twice with 500 µL of PBST (PBS containing 0.1% Tween-20) and incubated with BNIP3L antibody (1:100 diluted in PBST) for 30 minutes at room temperature. After removing the supernatant, the beads were washed twice with 500 µL of PBST and once with 400 µL of RIPA. Following this, the beads were incubated overnight at 4 °C in total protein extraction buffer, and then another round of washing was performed as described above. Finally, the beads were resuspended with 80 µL of SDS elution buffer and 20 µL of 5X loading buffer and boiled for 5 minutes. The resulting supernatant was transferred to a new EP tube for subsequent analysis.

### RNA interference (RNAi)

Double-stranded RNA (dsRNA) fragments targeting *RCOR1* (5’-GCAAAGUUGGAUGAAUACATT-3’) and *BNIP3L* (5’-UUCUCCGAACGUGUCACGUTT-3’) were designed and chemically synthesized (Sangon Bioteach, China). For transient knockdown experiments, the transfection mixture was prepared by mixing 200 μL of serum-free culture medium with 60 ng of dsRNA and 8 μL of siRNA Transfection Reagent (Polyplus, #101000036), according to the manufacturer’s instructions. HEK293 cells were incubated with the freshly prepared transfection mixture for a period of 48 hours under standard culture conditions to allow sufficient time for RNAi-mediated gene silencing, and then harvested and processed for subsequent biochemical analyses.

### Real-time quantitative PCR analysis

Total RNA was isolated from HEK293 cells transfected with EhPTP4 and BNIP3L dsRNA, respectively. The isolation was carried out using the E.Z.N.A. Total RNA Kit II (OMEGA, R6934-02) according to the manufacturer’s instructions. Subsequently, 1 μg of the total RNA was used to synthesize cDNA with the Hifair III 1st Strand cDNA Synthesis Kit (gDNA digester plus) (Yeasen, 11141ES). RT-qPCR was then performed using primers specific for *BNIP3L* (forward primer: AGGACAGAGTAGTTCCAGAGGCA, reverse primer: GGAATGTTTTCGGGTCTACTGG), *HMGA1* (forward primer: GGAAAAGGACGGCACTGAGA, reverse primer: TTAGGTGTTGGCACTTCGCT), *SLC5A3* (forward primer: ATGCTGCGGAATCCAACAGA, reverse primer: ACCCTCTGCACGATGACTTG) with *actin* (forward primer: CATGTACGTTGCTATCCAGGC, reverse primer: CTCCTTAATGTCACGCACGAT) as a control. The reaction program consisted of pre-denaturation at 98°C for 2 min, followed by denaturation at 98°C for 10 s, and annealing and extension for 20 s, for a total of 40 cycles. The results were analyzed using the LightCycler 96 (Roche, 05815916001).

Genomic DNA was isolated from HEK293 cells transfected with EGFP and BNIP3L, respectively. The isolation was carried out using the DNA Extraction Kit (Omega, D3396-02) according to the manufacturer’s instructions. *Eh-SSU* copies were analyzed through qPCR, the forward primer qF-*Eh-SSU* (5’- GAATGATTGAACAAGTTATTTTGAATGTG -3’) along with the reverse primer qR-*Eh-SSU* (5’-AACACGAAAGACTCAGACCTCTCA-3’). The standard plasmid constructed by *Eh-SSU* by gradient to establish a standard curve, to analyze the copy number of *E. hellem.*

### Yeast two-hybrid

The yeast two-hybrid experiment was conducted using the Y2HGold yeast strain. The strain was propagated and cultured under standard yeast culture conditions, followed by the preparation of competent cells using Yeastmake(tm) Yeast Transformation System 2 (Takara, #630439). Following transformation, the cells were resuspended in sterile 0.9% (w/v) sodium chloride solution (NaCl; Sangon Bioteach, A501218) and plated on SD medium lacking leucine and tryptophan (-Leu/-Trp). Plates were incubated at 30°C for 2–3 days to allow colony formation. Single colonies growing on the -Leu/-Trp plates were selected and cultured in 1.5 mL centrifuge tubes containing SD liquid medium lacking leucine and tryptophan (-Leu/-Trp) for 48 hours at 30°C with orbital shaking at 200 rpm. Cells were then pelleted by centrifugation, the supernatant was discarded and washed three times in 1 mL of sterile 0.9% NaCl solution to remove residual medium and metabolites. The cell pellet was resuspended in 300 µL of sterile 0.9% NaCl solution to prepare a uniform cell suspension. Subsequently, 10 µL aliquots were spotted onto SD/-Leu/-Trp/-His and SD/-Leu/-Trp/-His/-Ade selective plates containing X-α-Gal (Yeasen, #10903ES; 4 mg/mL). Plates were incubated at 30°C for 3–5 days to allow the development of colonies, confirming positive interactions.

### Mitophagy induction with CCCP

HEK293 cells were cultured in complete growth medium and transfected with the EhPTP4 expression vector for 42 hours. Following transfection, the culture medium was replaced with fresh medium containing the mitochondrial uncoupler CCCP (Selleck Chemicals, #S6494) at a final concentration of 20 μM. The cells were incubated under these conditions for an additional 6 hours to induce mitophagy. After treatment, the cells were harvested to extract proteins and perform Western blotting for BNIP3L and β-tubulin expression levels to evaluate mitophagy induction.

### Proteasome inhibition analysis

HEK293 cells were transfected with the EhPTP4 plasmid for 42 hours. Subsequently, the medium was replaced with fresh medium containing MG132 (MedChemExpress, HY-13259; 20 nM). After 6 hours, the cells were harvested, and total cellular proteins were extracted for Western blot analysis to determine BNIP3L and β-tubulin levels.

### Dual-luciferase reporter assay

HEK293 cells were plated in 12-well plates. The promoter-pGL3-basic vector, a recombinant plasmid constructed with a target promoter fragment approximately 900 bp upstream of the transcription start site (TSS) that had been amplified, was transfected into the cells at a dosage of 1 μg using the liposome transfection method. Simultaneously, either 1 μg of the EhPTP4 expression vector or 1 μg of the EGFP control vector was transfected. To normalize the transfection efficiency, 100 ng of the pRL-SV40 vector was added to each group as an internal reference. Forty-eight hours later, the cells were harvested, and the activity values of firefly luciferase and Renilla luciferase were measured using the dual-luciferase reporter gene detection system (Yeasen, 11402ES).

### Statistical analyses

The data were represented as mean values ± SEM for all statistical analyses using GraphPad Prism 9 software. The *p* values were determined by Student’s t-test of three independent biological experiments. Differences between two groups were considered statistically significant when *p* value was less than 0.05.

## Supporting information

S1 Fig Subcellular localization of EhPTP4 in RK13 cells infected with *E. hellem.* RK13 cells were infected with *E. hellem* for 48 hours. The cells were then fixed and immunostained using primary antibodies against EhPTP4, followed by an Alexa Fluor 488-conjugated anti-mouse-IgG secondary antibody (green). Nucleus were stained by 4’,6-diamidino-2-phenylindole (DAPI). N, nucleus; scale bar, 30μm.
(TIF)

S2 Fig Infection rate of HPSIME and HEK293 cells by *E. hellem.* Human primary small intestinal mucosal epithelial (HPSIME) cells (IMMOCELL, IMP-H006) and HEK293 cells were infected with Encephalitozoon hellem for 48 hours. Infected cells were quantified based on the presence of parasitophorous vacuole (PV). *, *p* < 0.05.
(PDF)

S3 Fig Catalytic activity of APEX2 in transgenic HEK293 cells. (A) Immunofluorescence assay was employed to analyze the catalytic activities of EhPTP4::APEX2 and EhPTP4^ΔHRD^::APEX2 in transgenic HEK293 cells. Blue signals indicated hoechst-labeled nuclei. The green signal represented the protein expressed by EGFP fusion (detected with antibody against HA). Red fluorescence showed biotin-labeled proteins (detected with Streptavidin-568). The figure shows the merged images. Bar scale: 10 μm. (B) Western blot analysis (using Anti-Streptavidin-HRP) was performed on transgenic HEK293 cells expressing EhPTP4::APEX2 and EhPTP4^ΔHRD^::APEX2 prior to the enrichment of biotin-labeled proteins with streptavidin beads. The “+” and “-” symbols denoted the presence or absence of APEX2 and biotin-phenol plus H_2_O_2_, respectively. (C) Western blot using streptavidin-HRP was carried out on the biotin-labeled proteins enriched with streptavidin beads. The “+” and “-” symbols indicated the presence or absence of APEX2 and biotin-phenol plus H_2_O_2_, respectively.
(TIF)

S1 Table Identification of proteins interacting with the EhPTP4. HEK293 cells were transfected with *EhPTP4::APEX2* and *EhPTP4*^*ΔHRD*^*::APEX2*, respectively. Biotinylated proteins were enriched using streptavidin magnetic beads and analyzed by LC-MS/MS. Following functional annotation and comparative analysis, four candidate proteins (GM2A, CNTNAP1, NCCRP1, and RCOR1) were found to be significantly enriched exclusively in *EhPTP4::APEX2*-transfected cells.
(XLSX)

S2 Table Quantitative proteome analysis of *EhPTP4*-transgenic HEK293 cells. The list includes 20 proteins significantly decreased in content.
(XLSX)

S3 Table Ubiquitinated proteome of *EhPTP4*-transgenic HEK293 cells. The list includes 1049 proteins which ubiquitination level significantly increased.
(XLSX)
