# Supplementary figures and images for: Microsporidian Encephalitozoon hellem inhibits host mitophagy by inducing ERAD to degrade BNIP3L
Source: PLoS Pathog. 2026 Mar 23;22(3):e1014078. doi: 10.1371/journal.ppat.1014078 (PMC13029686; doi:10.1371/journal.ppat.1014078)

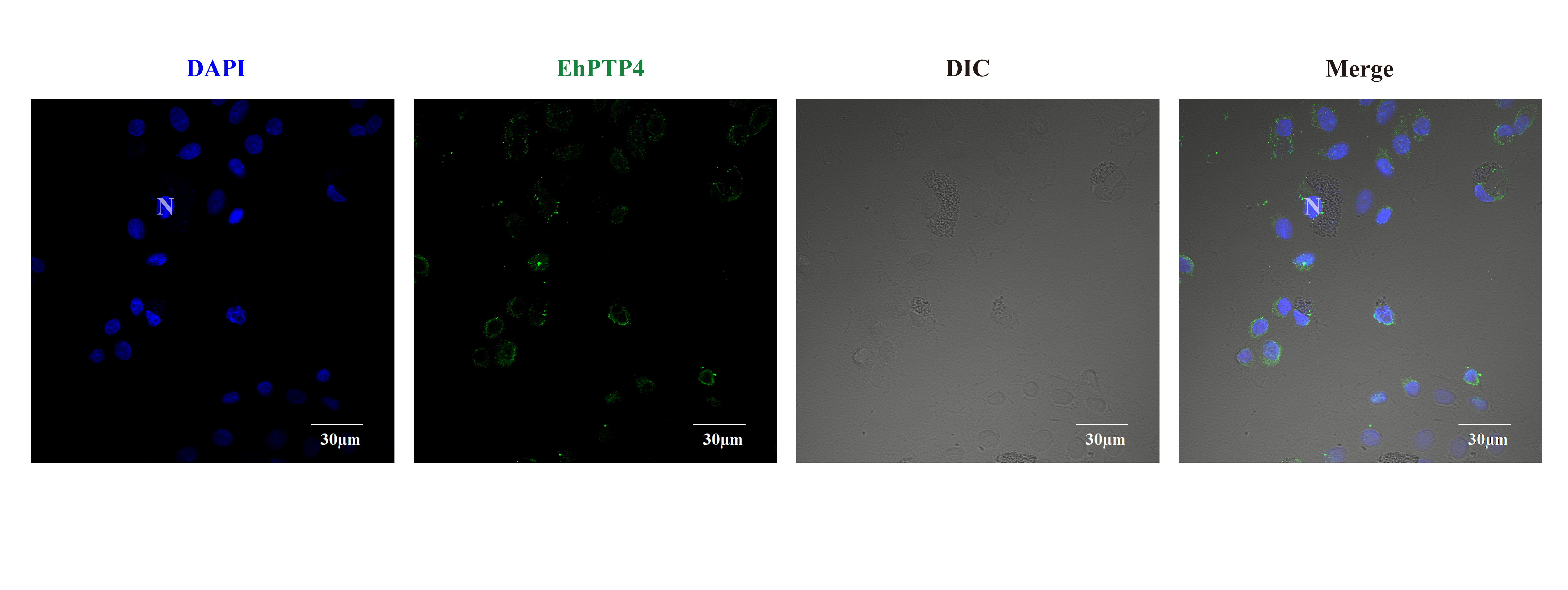

Supplement: S1 Fig — RK13 cells were infected with E. hellem for 48 hours. The cells were then fixed and immunostained using primary antibodies against EhPTP4, followed by an Alexa Fluor 488-conjugated anti-mouse-IgG secondary antibody (green). Nucleus were stained by 4’,6-diamidino-2-phenylindole (DAPI). N, nucleus; scale bar, 30μm. (TIF) [file ppat.1014078.s001.tif]

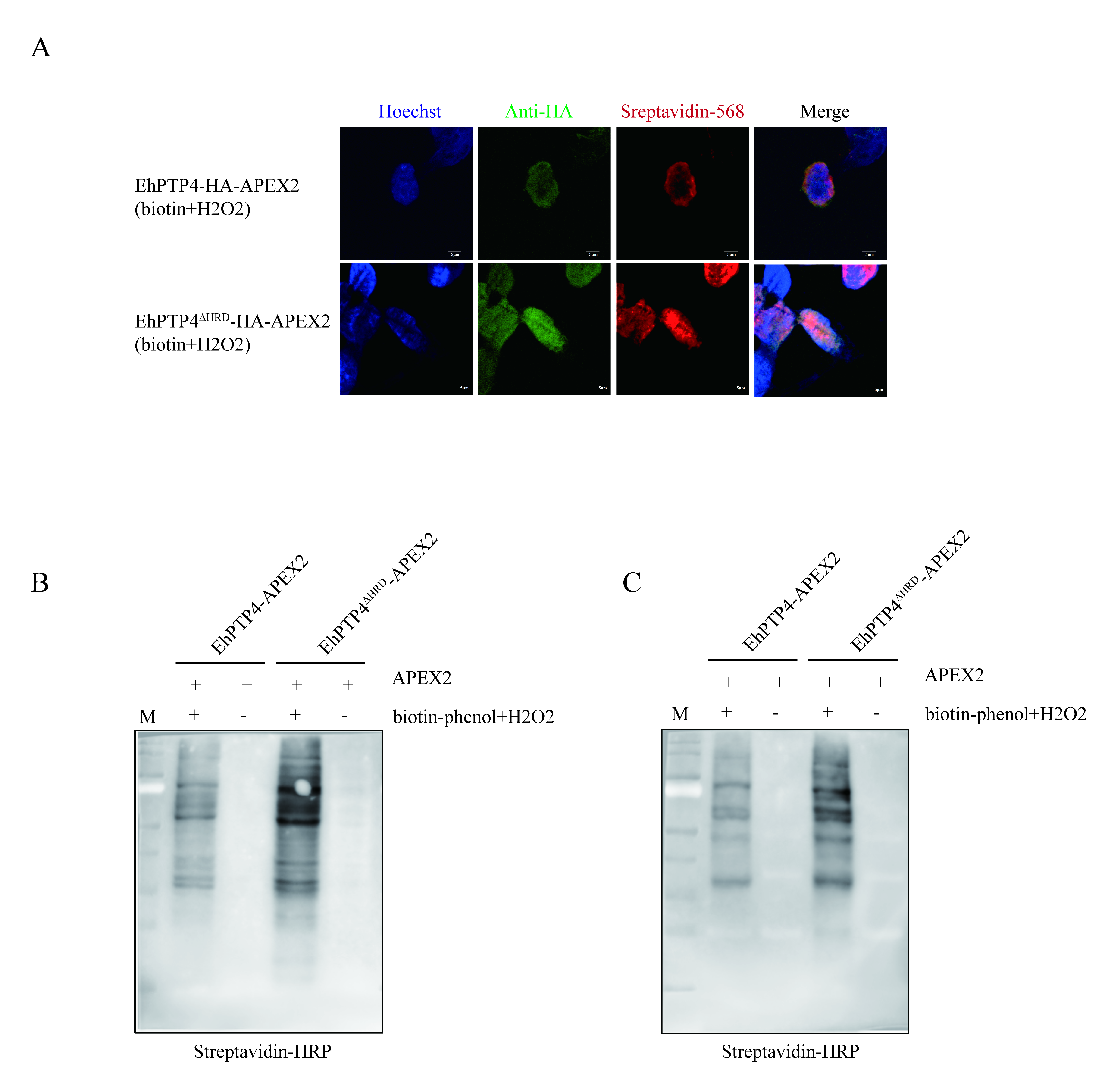

Supplement: S3 Fig — (A) Immunofluorescence assay was employed to analyze the catalytic activities of EhPTP4::APEX2 and EhPTP4ΔHRD::APEX2 in transgenic HEK293 cells. Blue signals indicated hoechst-labeled nuclei. The green signal represented the protein expressed by EGFP fusion (detected with antibody against HA). Red fluorescence showed biotin-labeled proteins (detected with Streptavidin-568). The figure shows the merged images. Bar scale: 10 μm. (B) Western blot analysis (using Anti-Streptavidin-HRP) was performed on transgenic HEK293 cells expressing EhPTP4::APEX2 and EhPTP4ΔHRD::APEX2 prior to the enrichment of biotin-labeled proteins with streptavidin beads. The “+” and “-” symbols denoted the presence or absence of APEX2 and biotin-phenol plus H2O2, respectively. (C) Western blot using streptavidin-HRP was carried out on the biotin-labeled proteins enriched with streptavidin beads. The “+” and “-” symbols indicated the presence or absence of APEX2 and biotin-phenol plus H2O2, respectively. (TIF) [file ppat.1014078.s003.tif]
