# Supplementary material for: Microsporidian Encephalitozoon hellem inhibits host mitophagy by inducing ERAD to degrade BNIP3L
Source: PLoS Pathog. 2026 Mar 23;22(3):e1014078. doi: 10.1371/journal.ppat.1014078 (PMC13029686; doi:10.1371/journal.ppat.1014078)
Supplement: S2 Fig — Human primary small intestinal mucosal epithelial (HPSIME) cells (IMMOCELL, IMP-H006) and HEK293 cells were infected with Encephalitozoon hellem for 48 hours. Infected cells were quantified based on the presence of parasitophorous vacuole (PV). *, p < 0.05. (PDF) [file ppat.1014078.s002.pdf]

HEK293 cells infected by *Encephalitozoon hellem*

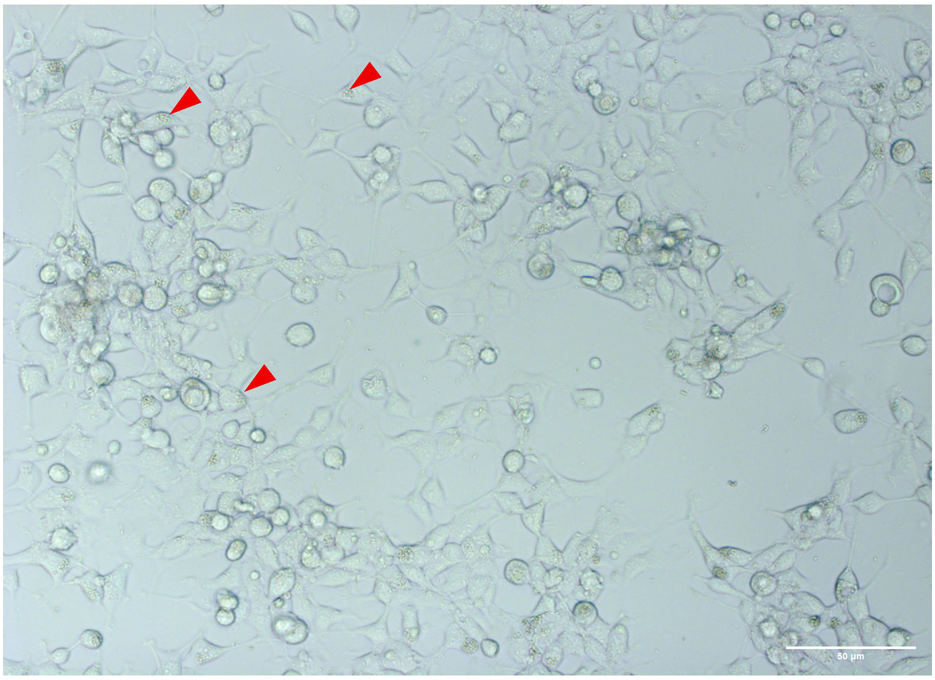

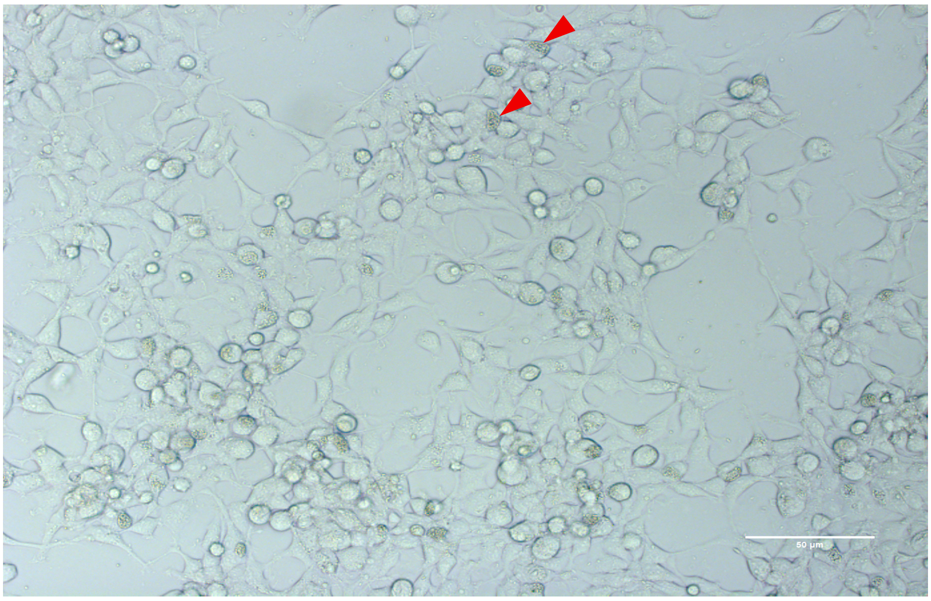

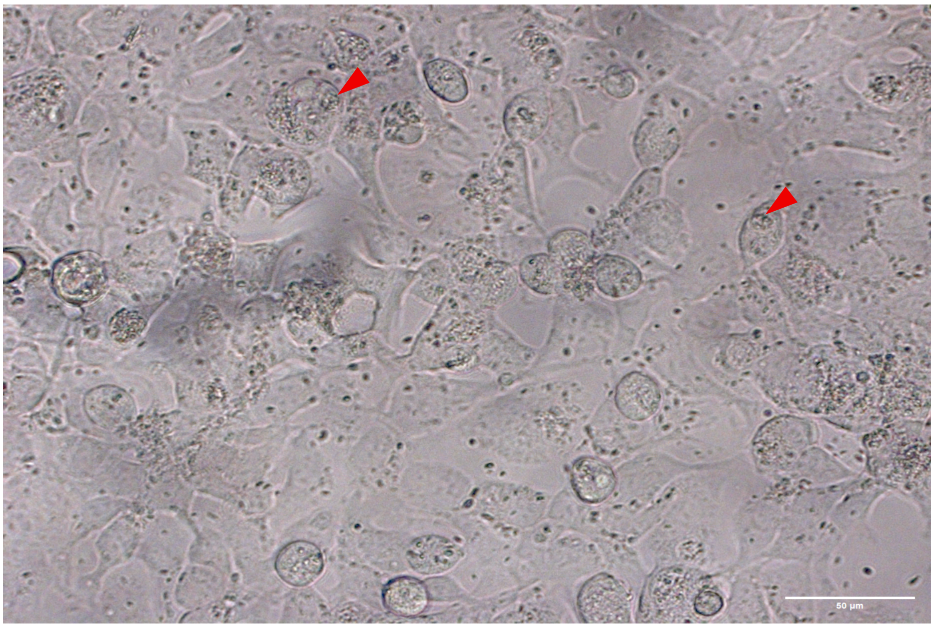
50 µm

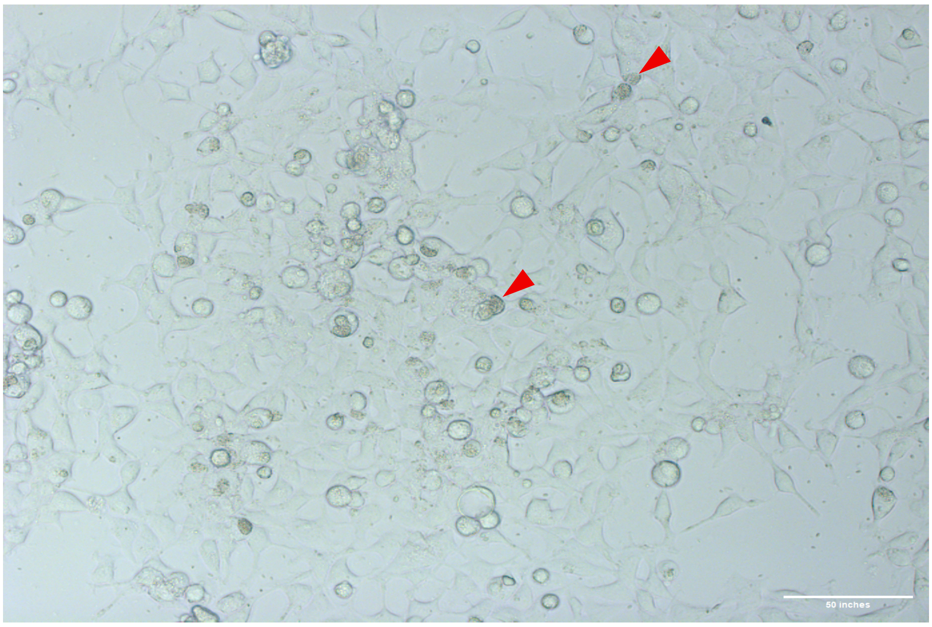
50 inches

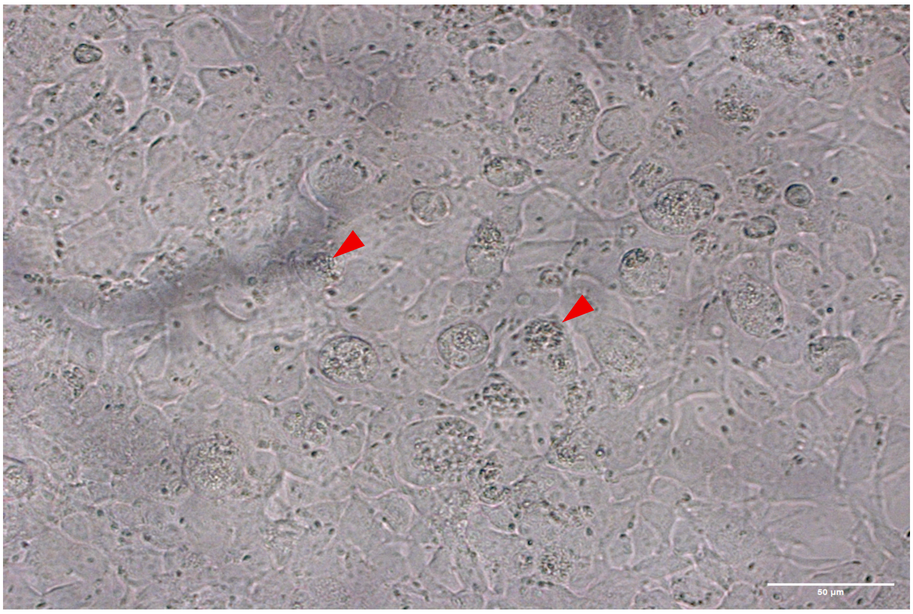
50 μm

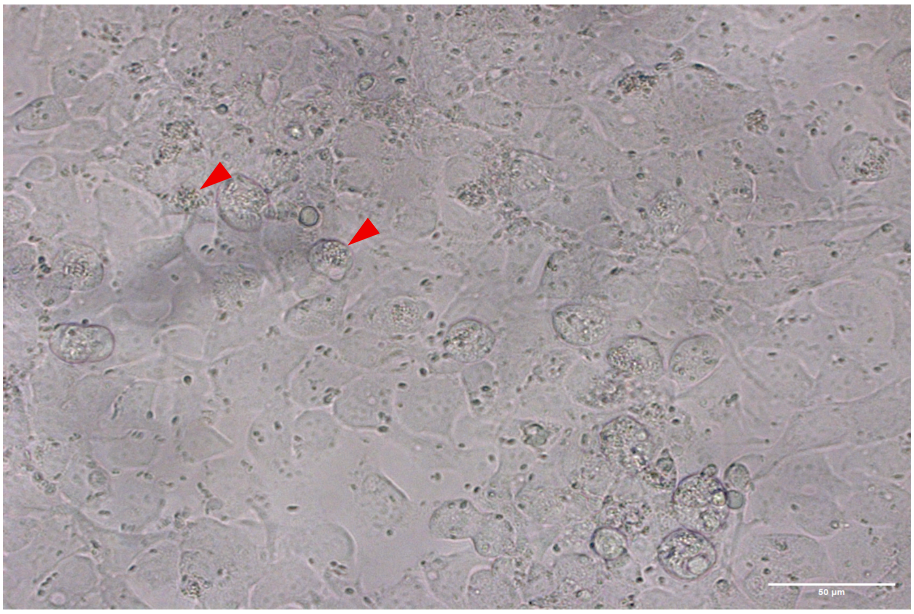
50 μm

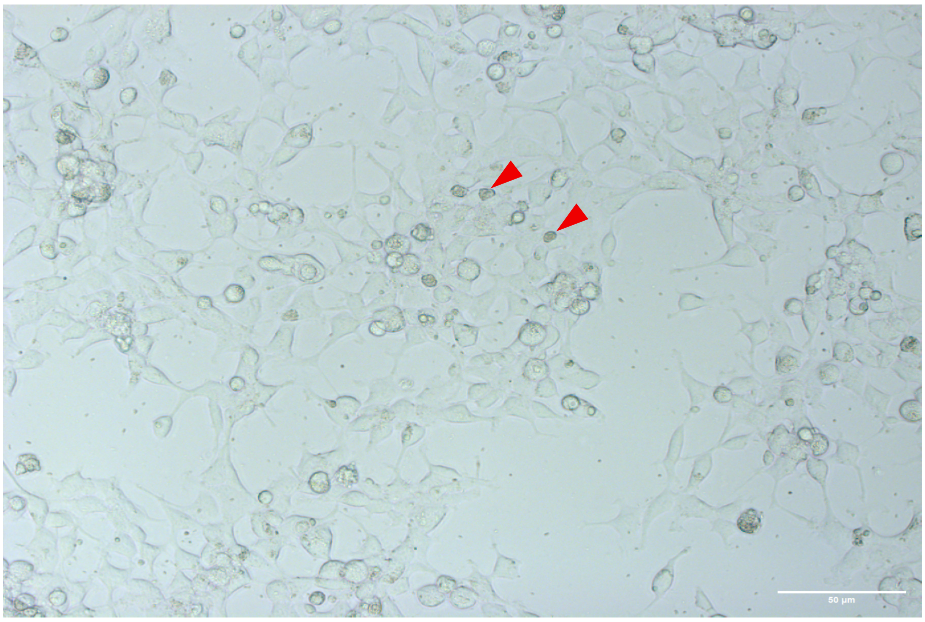
50 µm

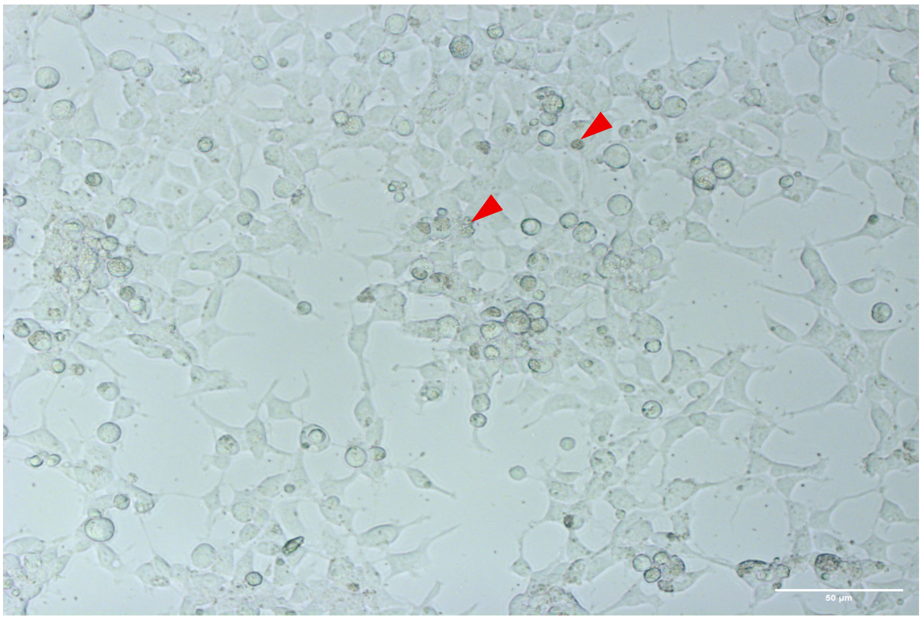
50 µm

HPSIME cells infected by *Encephalitozoon hellem*

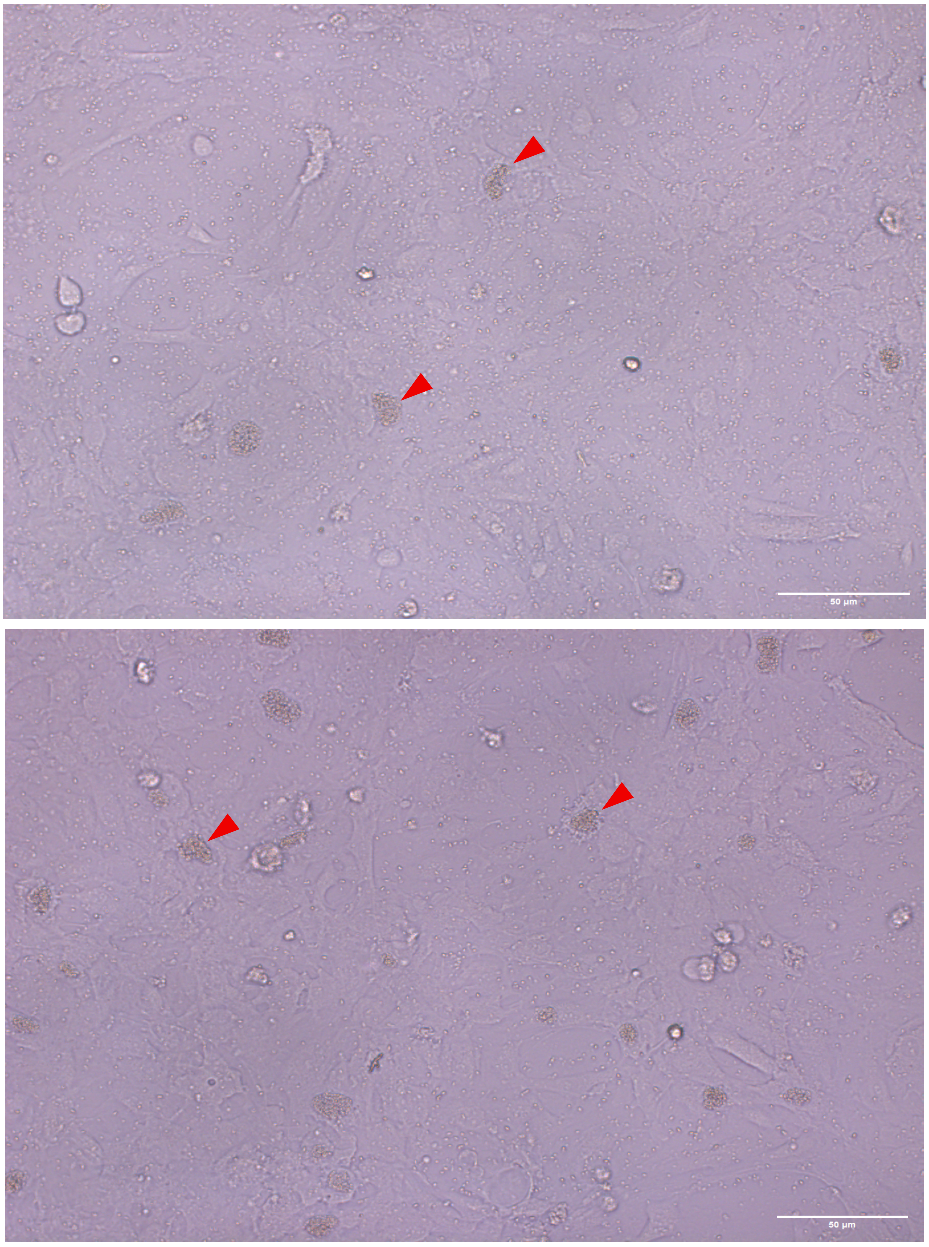

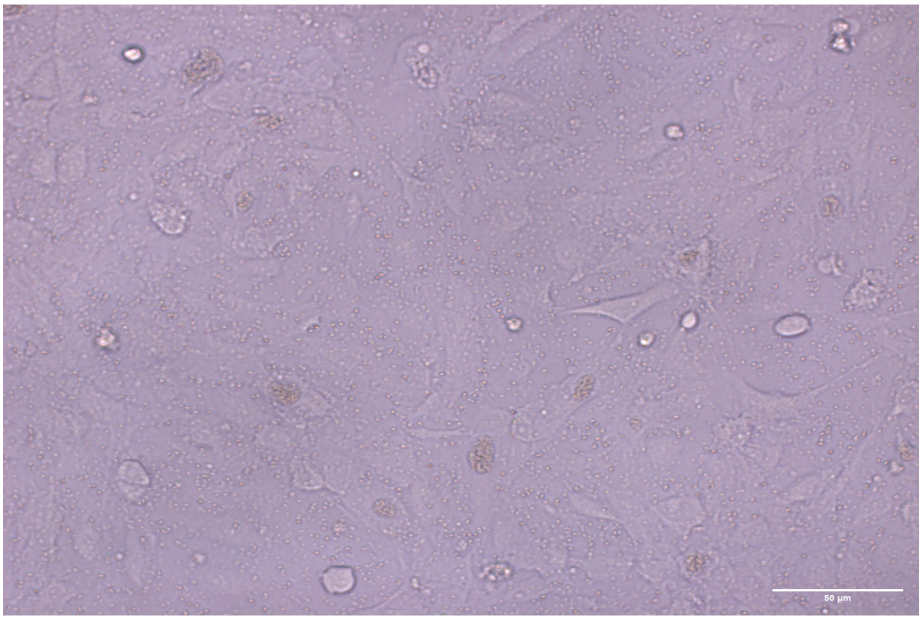
50 µm

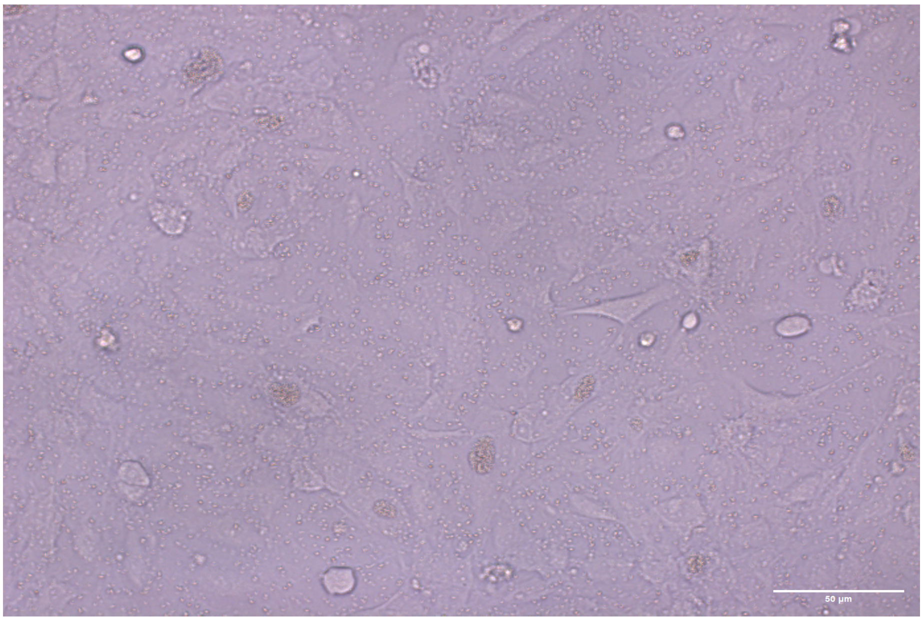
50 µm

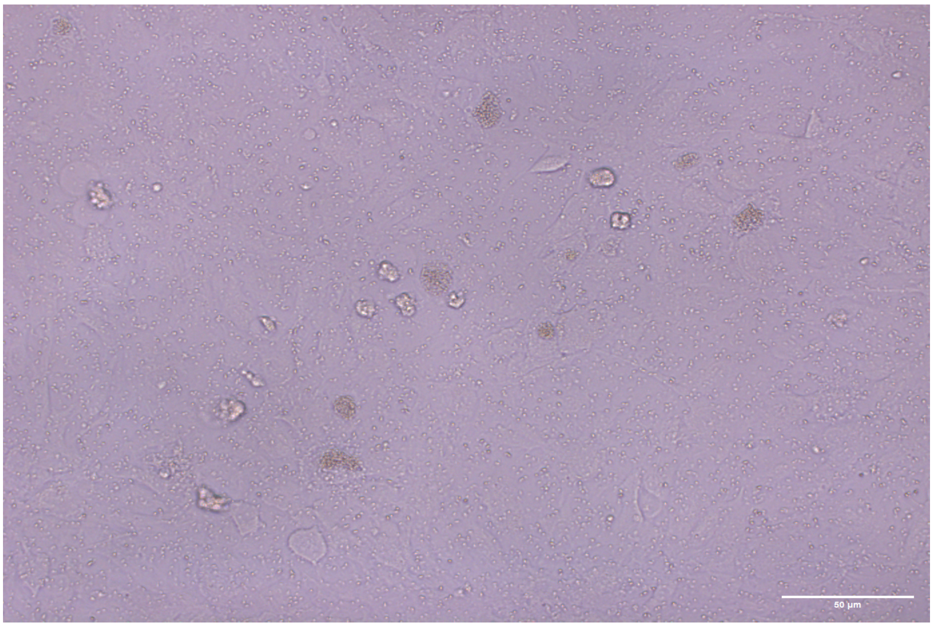
50 µm

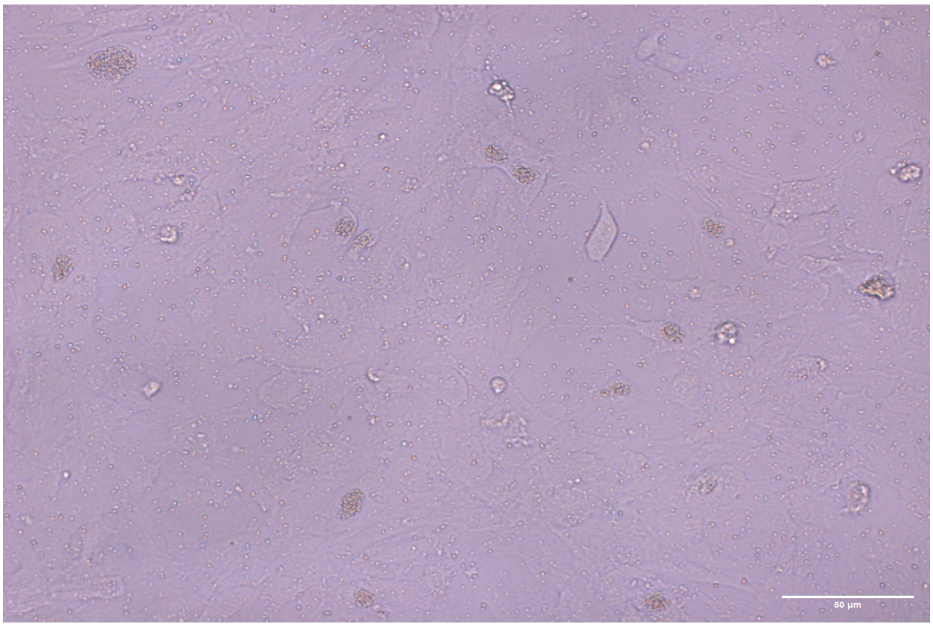
50 µm

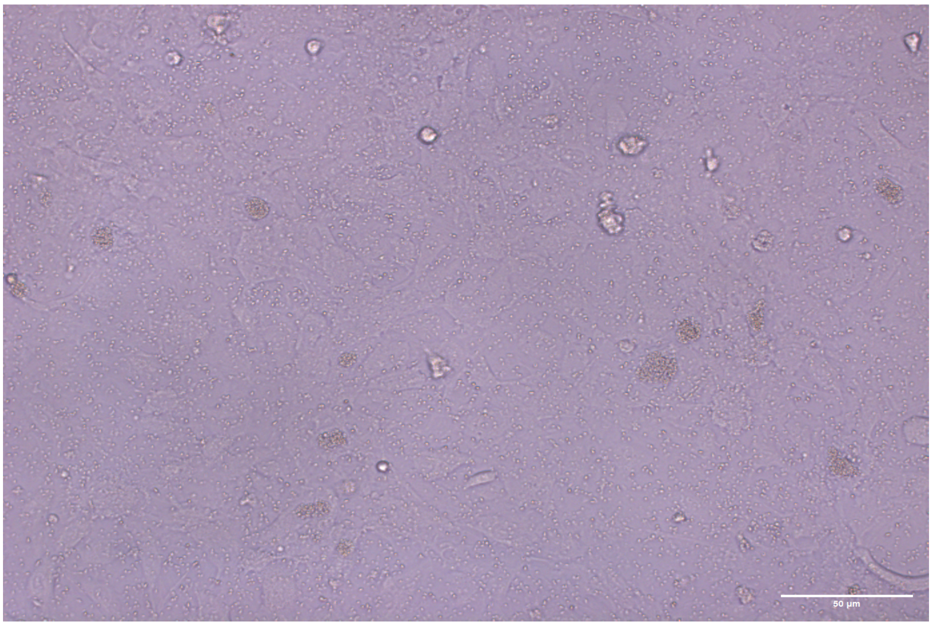
50 μm

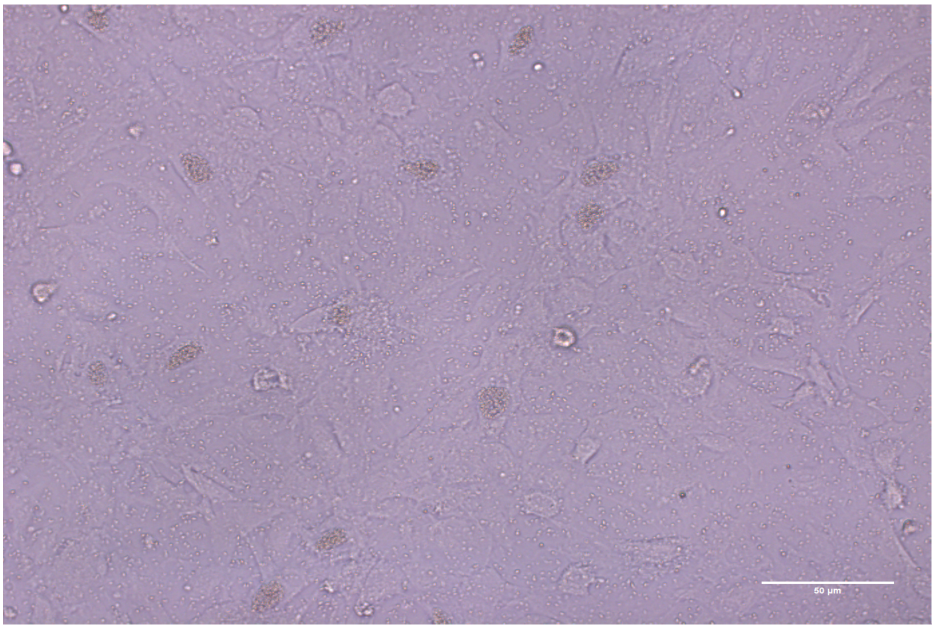
50 μm

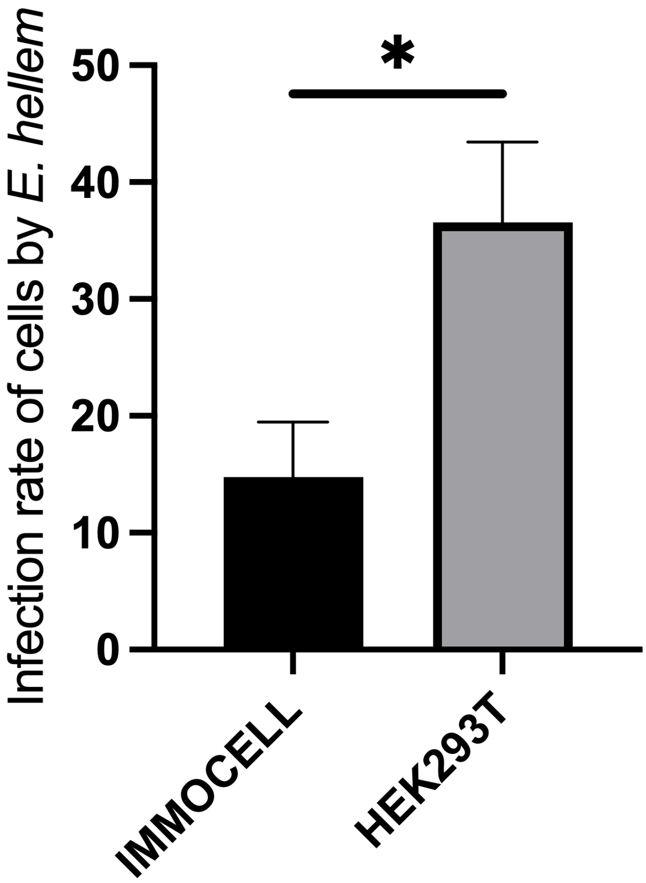

Figure S3. Infection rate of HPSIME and HEK293 cells by *E. hellem*.

Human primary small intestinal mucosal epithelial (HPSIME) cells (IMMOCELL, IMP-H006, http://www.immocell.com/?rydxb-13/7582.html) and HEK293 cells were infected with *Encephalitozoon hellem* for 48 hours. Infected cells were quantified based on the presence of parasitophorous vacuole (PV). *, $p < 0.05$.
